# Overview of Inorganic Electrolytes for All-Solid-State Sodium Batteries

**DOI:** 10.1021/acs.langmuir.4c01845

**Published:** 2024-07-30

**Authors:** Aakash Carthick Radjendirane, Dheeraj Kumar Maurya, Juanna Ren, Hua Hou, Hassan Algadi, Ben Bin Xu, Zhanhu Guo, Subramania Angaiah

**Affiliations:** † Electro-Materials Research Laboratory, Centre for Nanoscience and Technology, Pondicherry University, Puducherry 605 014, India; ‡ College of Materials Science and Engineering, Taiyuan University of Science and Technology, Taiyuan 030024, China; § Integrated Composites Laboratory (ICL), Department of Mechanical and Construction Engineering, Northumbria University, Newcastle Upon Tyne NE1 8ST, U.K.; ∥ Department of Electrical Engineering, Faculty of Engineering, Najran University, Najran 11001, Saudi Arabia

## Abstract

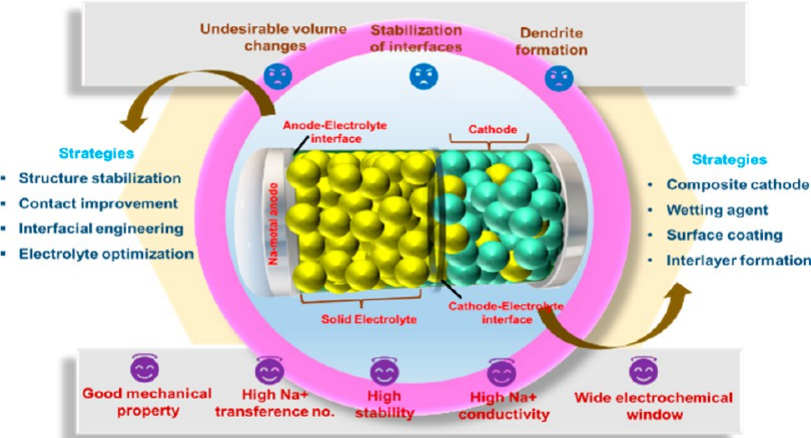

All-solid-state sodium batteries (AS^3^B) emerged as a strong contender in the global electrochemical energy storage market as a replacement for current lithium-ion batteries (LIB) owing to their high abundance, low cost, high safety, high energy density, and long calendar life. Inorganic electrolytes (IEs) are highly preferred over the conventional liquid and solid polymer electrolytes for sodium-ion batteries (SIBs) due to their high ionic conductivity (∼10^–2^–10^–4^ S cm^–1^), wide potential window (∼5 V), and overall better battery performances. This review discusses the bird’s eye view of the recent progress in inorganic electrolytes such as Na-β”-alumina, NASICON, sulfides, antipervoskites, borohydride-type electrolytes, etc. for AS^3^Bs. Current state-of-the-art inorganic electrolytes in correlation with their ionic conduction mechanism present challenges and interfacial characteristics that have been critically reviewed in this review. The current challenges associated with the present battery configuration are overlooked, and also the chemical and electrochemical stabilities are emphasized. The substantial solution based on ongoing electrolyte development and promising modification strategies are also suggested.

## Introduction

The surge in energy consumption, highly depleting reserves of fossil fuels^1^ and rapidly increasing pollution,^2^ has prompted researchers to develop highly efficient renewable energy technologies.^3−5^ In the present scenario, an immense amount of attention has been paid to technological advancements for energy conversion and storage devices.^6−8^ The inevitable demands of utilizing renewable energy storage devices such as batteries and supercapacitors hold a promise for energy supply in hybrid electrical vehicles and portable electronics.^9−12^ All-solid-state sodium batteries (AS^3^B) have become ubiquitous by replacing current state-of-the-art Li-ion batteries (LIB) owing to their better performance, similar electrochemistry, safer operation, substantial energy density, and lower cost.^13−16^

**Figure 1:**
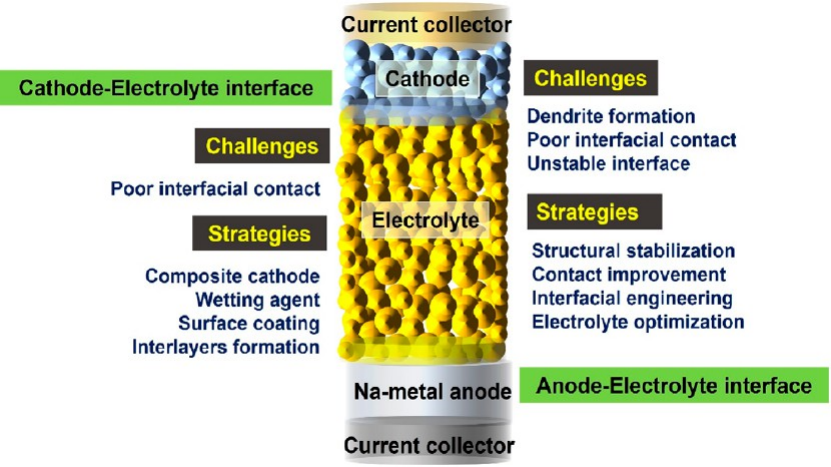
Interfacial challenges and strategies for inorganic electrolytes in AS^3^B.

In comparison to the counterpart lithium metal,^17^ sodium metal inherits a similar electrochemical characteristic (i.e., a specific capacitance value of 1166 mA h g^–1^ and a standard reduction potential of −2.71 V versus a standard hydrogen-based electrode). In contrast, sodium metal is more chemically reactive toward conventional organic liquid electrolytes, causing safety hazards such as short circuits due to the inevitable formation of dendrites.^18−21^ In the current context, AS^3^B employing solid-state electrolytes (SSEs) extensively addresses the safety hazards and enables the reliable utilization of a high electrochemical potential window containing cathode materials and metal-based anodes contributing to ultralong operation and excellent energy density. Trending AS^3^B demonstrates significant characteristics such as high ionic conductivity (∼10^–4^ to 10^–3^ S cm^–1^), negligible electronic conductivity (∼10^–12^ S cm^–1^), low self-discharge, high thermal stability, variable working temperature, a wide electrochemical potential window, and inertness toward shocks and vibrations as shown in Figure 1. A typical configuration of AS^3^B consists of a Na metal anode, SSE, and an electrolyte-contained cathode.^22,23^ As the critical component of AS^3^B, the solid electrolyte makes an indispensable contribution by serving as both an excellent ionic conductor and a separator between the electrodes. Primarily, the SSEs are categorized into inorganic electrolytes, polymer electrolytes, and inorganic-polymer composite electrolytes.^24−28^

In general, inorganic electrolytes for AS^3^B are very safe and reliable as they inhibit dendrite formation due to their absence of leakage. They offer high thermal stability, a nonvolatile nature, and ease of design, whereas liquid electrolytes may rapidly catch fire in nonaqueous-based solvents. Furthermore, they also exhibit high ionic conductivity (10^–2^ to 10^–3^ S cm^–1^) and a wide electrochemical stability window (∼5 V) with a high energy density. Solid polymer electrolytes, on the other hand, have low ionic conductivity (10^–4^ to 10^–6^ S cm^–1^) and and a small electrochemical stability window (ESW, ∼3.4 V) due to their high ionic transfer resistance. IEs show highly stable structures with a wide temperature range (−70 to 500 °C), whereas high interfacial resistance was developed between electrolyte layers for the solid as well as composite polymer electrolytes (CPE). Similarly, the aggregation of inorganic particles over a polymer has a greater disadvantage for solid CPE. Overall, the inorganic electrolytes lead to higher storage life and better battery performance compared to all other solid-state electrolytes.^29,30^

This review mainly focuses on the progress of inorganic electrolytes for AS^3^B. There are several review papers in this domain. However, their primary focus is on the development and categorization of inorganic materials. Hence, recent advances in improving the ionic conductivity, ESW of ISE, and electrode–electrolyte interface mechanism have not yet been reviewed.^5,26^ Herein, we have summarized solely the development of inorganic electrolytes by emphasizing their classifications and respective mechanisms for ionic conduction. Additionally, strategies to enhance recent progress in the improvement of the interfacial characteristics, which include cathode–electrolyte and anode–electrolyte aspects, are critically assessed. Furthermore, the mechanism of dendrite formation and the possible solution are also addressed. The current challenges associated with the present battery configuration involve emphasizing the chemical and electrochemical stabilities. A substantial solution based on the ongoing electrolyte development and promising modification strategies is also suggested.

## Bottleneck of All-Solid-State Sodium Batteries

### Undesirable Volume Changes during Plating and Stripping Processes

It is noteworthy that a traditional intercalation-type electrode such as graphite undergoes a 10% volume change to accommodate the ions on the anodic side during the intercalation and deintercalation processes. Current AS^3^Bs employed metallic sodium as the anode that undergoes a much greater volume change compared to the graphite during the electrochemical plating and stripping process. This uncontrollable volume expansion led to the destruction and reformation of the solid electrolyte interfacial layer at the anode side upon successive insertion and disinsertion of Na^+^ in AS^3^Bs. Thus, inevitable reversible volume exchange during the plating/stripping processes led to the induction of mechanical stress, which in turn led to the electrochemical pulverization of the electrode material. This adversely contributes to the capacity fading in current AS^3^Bs. Comprehensive effort is directed toward the mitigation of undesirable volume expansion for the long-term cyclic operation in AS^3^Bs.

### Stabilization of Interfaces

In AS^3^B, achieving a stable solid–solid interface between the Na metal and inorganic ceramic electrolyte is a major hurdle in the realization of electrochemically stable and high-performing energy storage. The Na metal, being highly reactive, effectively reduces the IEs and consequently destabilizes the Na–IEs interface, degrading the device performance. Particularly in AS^3^B, an ideal solid electrolyte interface (SEI) must inherit the following characteristics:

- SEI should be highly ionically conductive and act as an electronic insulator across the electrode–electrolyte interface to ensure uniform Na^+^ deposition and thereby reduce the preferential Na deposition.
- SEI should be electrochemically and chemically inert to prevent undesirable chemical reactions with the electrolyte deteriorating the electrochemical performance.
- SEI should be mechanically robust to ensure successive volume change and dendrite propagation during the deposition and stripping process in AS^3^B.

Inevitable subpar electrochemical cyclic performance and low Columbic efficiency due to unstable SEI formation at the electrode–electrolyte interface are effectively addressed by introducing intimate contact across the interface. Intimacy of contact at the interface is assessed by measuring the area-specific resistance (ASR) that substantially ensures the homogeneous stripping and deposition of Na^+^ ions during the operation in AS^3^B. The ASR across the sodium metal and IEs can be determined using Equation 1
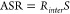
1where ASR, *R*_*inter*_, and *S* refer to the area-specific resistance, internal resistance offered by the fabricated cell, and effective contact area of the metallic Na–IE interface, respectively. The effective contact area in the area-specific resistance (ASR) refers to the interface between the inorganic electrolyte and the electrode. The contact area is a crucial factor in reducing the ASR. A lower ASR leads to better battery performance. In this regard, a larger contact area allows for better electron and ion transfer between the inorganic electrolyte and the electrode, leading to lower resistance and enhanced battery efficiency. Also, reaching an ASR_electrolyte_ of 24 Ω cm^2^ is the highest ASR_cell_ value for commercial 18650LIBs.^31^ In the case of the symmetric cell configuration where the same contact area is involved in ASR, the measured *R*_*inter*_ is halved to evaluate the ASR value. A large value of ASR results in an increasingly high overpotential that ultimately reduces the energy efficiency and dendrite formation. This adversely leads to the inhomogeneous nucleation in the Na^+^ ions in the plating process and thus increases the local current density. Therefore, developing a good interface between the electrode and the inorganic electrolyte, optimizing the electrode–electrolyte interface, and reducing the space between the electrode and the electrolyte can help to reduce the interfacial resistance and improve the battery’s performance.

### Dendrite Formation at the Metal Anode

The dendritic growth of Na metal in SSE is due to volume change, chemical instability, and mechanical incompatibility. In general, the dendrite growth in a liquid electrolyte system occurs when Na metal undergoes directional dendrite formation. Apparently, the LUMO level of the liquid electrolyte is below the redox potential of Na, which reductively decomposes the electrolyte until the formation of a solid electrolyte interface (SEI) layer. In contrast, dendrite growth in SSE undergoes two mechanisms. The first is similar to the liquid electrolyte—directional dendrite formation—and the latter is the dispersed Na dendrite plating within the SSE. The plating of Na ions occurs in high-energy areas such as grain boundaries, voids, and defects within the SSE. Also, when diffusing through the SSE, the Na^+^ ions are reduced by the leaking electrons from these areas (Na^+^ → Na^0^). Thus, dendrites grow and fill up the pores in subsequent cycles. After all of the pores are filled up, the dendritic growth commences at the grain boundary, leading to a detrimental effect on the battery operation. However, compositional elements of some inorganic SSE decrease with the Na metal to form SEI layers (e.g., Na_2_O, NaI, NaCl, etc.). These layers suppress the contact between the metal anode and high energy areas of inorganic SSE, reducing the dendrite growth.^32^

Furthermore, the sodium metal inherently shows a higher chemical reactivity and weaker mechanical structures. This is due to the larger atomic radius and weaker metallic bonding in sodium metal than those of the lithium metal. Like the conventional LIBs, AS^3^Bs are plagued by the growth of dendrite upon successive electrochemical plating and striping processes, thus giving birth to safety hazards. The nonuniform deposition at metallic sodium led to the formation of sodium dendrites and cavity formation. From the thermodynamical concept, the formation of dendrites occurs when the electrochemical potential is lower than the standard electrode potential of metals (−2.71 V vs SHE for Na/Na^+^). Consequently, conventional Na-ion batteries rarely encounter the issue of dendrite formation because of the higher working potential of intercalation-based anodes (e.g., 2.84 V vs SHE for graphite) compared to that of metals. The possible causes of the dendrite’s formation in conventional metal-ion batteries are the concentration profile influence of standard organic liquid electrolyte with sodium metal, the instability of the plane interface, and nonuniformity in the electrode–electrolyte interface. Herein, the term “plane interface” refers to the boundary or contact surface between two different materials present within the battery system. These interfaces play a critical role in determining the fundamental aspects of electron transfer and ion transport, which are essential to the overall performance of the battery. One possible cause of dendrite formation is the instability of the plane interface, which can be influenced by the mass transport of Na ions in the liquid electrolyte and the SEI layer. If the mass transport is nonuniform, then it can lead to an uneven deposition of Na. Additionally, the SEI layer can be unstable and can undergo continuous formation and dissolution, leading to the formation of dendrites. These are the factors causing the formation of dendrites in AS^3^B.

## Ionic Conduction Mechanism

The pursuit of high ionic conductivity renders the development of highly ionically conductive IEs. A preferred IE must be an ionic conductor inheriting negligible electronic conductivity. Extensive effort has been expended in the exploration of advanced electrolyte materials and the modification strategies (i.e., doping, substitution, composite formation, coating, crystal formation, ceramization, etc.) to achieve high ionic conductivity. In IEs, ionic conduction originates through the long-range transport of mobile sodium ions via interstitial hopping with a similar energy state. Intrinsic ionic conduction relies on the availability of mobile ions/voids, the hopping vacancy sites, and the concerned energy barrier for the hopping of mobile ions. The ionic conduction is extensively influenced by the crystal structure, lattice dynamics grain boundaries, and defect structure. The responsible mechanism in IEs is observed mainly in three ways: (1) vacancy diffusion, (2) interstitial site migration, and (3) knock-on or correlation-based movement through grain boundaries. The ionic conductivity (σ) of IEs is governed by the Arrhenius equation as shown in Equation 2:
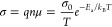
2As evident from the above equation, σ is the product of charge (*q*), concentration of mobile ions (*n*), and ionic mobility (μ) of the charge carrier of an IE. Ionic conduction in a thermally activated process that obeys the typical Arrhenius equation, in which *k*_*B*_ refers to the Boltzmann constant, *T* refers to temperature, and *E*_*a*_ refers to the characteristic activation energy required for ion conduction. In the case of IEs, high concentration of mobile ions (*n*) and lower activation energy (*E*_*a*_) are the crucial factors in achieving high ambient temperature σ.^33^

### Ionic Conduction Mechanism of Oxide-Based Electrolytes

**Figure 2:**
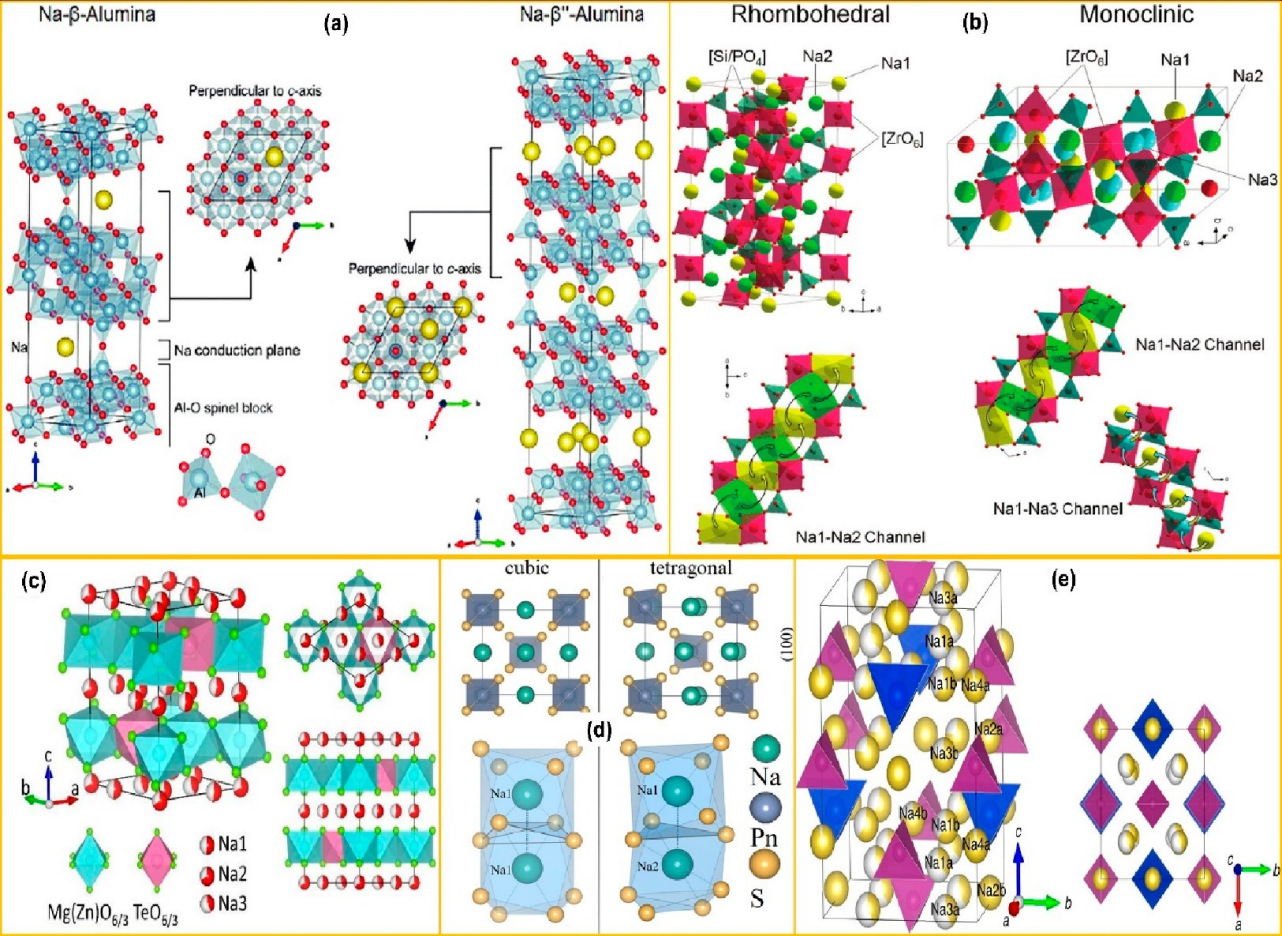
Crystal structures of (a) Na-β/β′′-Al_2_O_3_. Panel a is reproduced from ref (45). Copyright 2016 Elsevier Ltd. (b) Rhombohedral and monoclinic polymorph of Na_3_Zr_2_Si_2_PO_12_ (NASICON). Panel b is reproduced with permission from ref (22). Copyright 2018 Wiley VCH Gmbh. (c) P-2-type layered electrolyte. Panel c is reproduced from ref (40). Copyright 2018 American Chemical Society. (d) Cubic and tetragonal phases of Na_3_PnS_4_. Panel d is reproduced with permission from ref (46). Copyright 2020 American Chemical Society. (e) Structure of Na_10_SnP_2_S_12_. Panel e is reproduced from ref (47). Available under a CC-BY 4.0 license. Copyright 2016 Richards et al.

The ionic transport mechanism of Na-β-Al_2_O_3_ is stated on account of its crystal framework is shown in Figure 2a. It is evident from the chemical structure that Na-β′′-Al_2_O_3_ is composed of basic building blocks of alternatively arranged layers with spinel blocks and a conduction plane. Specifically, the spinel block is composed of four layers of stacked oxygen ions surrounded by aluminum ions accompanied by the conduction planes comprising packed oxygen and sodium ions. In the case of its two polymorphs (i.e., Na-β′′-Al_2_O_3_ and Na-β-Al_2_O_3_), the ionic conduction occurs solely through the conduction plane and there is a negligible contribution of vertical stacks. In fact, both β- and β″-alumina formed the tetrahedral and octahedral Al–O spinel blocks. In particular, the β-phase has a hexagonal lattice representing the space group *P*6_3_/*mmc*, whereas the β″-phase has a rhombohedral lattice with a space group of *R*3̅*m*. The unit cell of the β-phase contains one Na ion, whereas the β″-phase has two Na ions in each of the conduction layers. Therefore, Na-β′′-Al_2_O_3_ (0.35 S cm^–1^) with a higher Na^+^ concentration and a larger unit cell with dissimilar oxygen stacking sequences serves as a better ionic conductor than Na-β-Al_2_O_3_ (0.3 S cm^–1^).^34^

Similarly, NASICON’s structural framework plays a vital role in facilitating appreciable ionic transport. NASICON comprises a covalent three-dimensional framework structure with corners shared by MO_6_ octahedra and PO_4_ tetrahedra that constitute a skeletal structure with a high availability of interstitial sites as 3D interconnected Na-ion migration pathways, as well shown in Figure 2b. NASICONs can form three types of crystal structures (i.e., rhombohedral, monoclinic, and triclinic based on composition).^35^ In the rhombohedral crystal structure of Na_1+*x*_Zr_2_Si_*x*_P_3_O_12_ (0 ≤ *x* ≤ 3), Na^+^ resides on two sites for *x*< 0. The large number of mobile Na^+^ and the adjacent vacancies can coexist in the rhombohedral phase, which is very beneficial for Na^+^ diffusion. At the same time, a deformed monoclinic phase would form a less symmetrical structure that may be conducive to Na^+^ migration. The migration of Na^+^ occurs from Na(1)–Na(2) channels via successive ion migration, as shown in Figure 2b. On the other hand, the splitting of Na(2) sites into another Na(3) site takes place in the monoclinic phase. Consequently, four bottleneck channels are created (i.e., two from Na(1)–Na(2) and the other two from Na(1)–Na(3)).^35^

In case of P-2-type layered materials (i.e., Na_2_Cu_2_TeO_6_, Na_2_Ni_2_TeO, Na_2_Zn_2_TeO, and Na_2_Mg_2_TeO_6_ etc.), there exists a P-2 type Na_*x*_CoO_2_ structure with a complete M^2+^/Te^6+^ ordering in each (MO_2_)_*n*_ layer.^36,37^ These P-2-type layered structures exists in different polymorph hexagonal space groups (i.e., *P*6_3_/*mcm* (Na_2_Ni_2_TeO_6_) and *P*6_3_22 (Na_2_Zn_2_TeO_6_/ Na_2_Co_2_TeO_6_)) based on the in-plane shift of the (M_2_Te)_*n*_ layers.^38^ Basically, there are three crystallographic sites evident from the quantitative analysis of X-ray diffraction, where Na^+^ resides in Na_2_M_2_TeO_6_ (i.e., Na1(6g), Na2(2a), and Na3(4f)). Theoretical studies such as molecular dynamics (MD) simulation on the Na^+^ ion transport in Na_2_Ni_2_TeO_6_ revealed that the hopping of Na^+^ ions occurs dominantly through Na1 to Na2, as compared to the Na3 site, as shown in Figure 2c. This is due to the fact that Na3 crystallographic sites (−2.32 eV) have a higher potential energy than Na1 and Na2 sites (−2.45 and −2.65 eV, respectively).^39^ Additionally, the bond valence sum (BVS) studies stated that Na3 inherits the same potential energy values as Na1 and Na2 in Na_2_Zn_2_TeO_6_ due to the lesser repulsion of Na^+^ with Zn^2+^ in the NZTO framework than that of Na^+^ with Te^6+^ in Na_2_Ni_2_TeO_6_.

In the case of the layered oxides, the larger spacing of Na_2_Zn_2_TeO_6_ is due to the closest oxygen atoms in adjacent layers, which share the same (*x*, *y*) coordinates. The important factor is mainly the O–O distance rather than the layer separation, and they are found to be 3.42 and 3.58 Å for P2 and O′3, respectively. The shorter O–O distance in the P2-type Na_2_Zn_2_TeO_6_ causes additional repulsion between the layers and increases the ionic conductivity. NZTO has a capability to enlarge the space between the strongly bonded 2D layers as shown in Figure 2c. In this way, a greater number of Na^+^ ions gain a spacious interstitial migration path comfortably than the equivalent close-packed inorganic oxide-based electrolytes. Considerable attention has been paid to the insight into the intercalation and diffusion of Na ions existing in the lamellar oxide system. Among all of the investigated Na_2_M_2_TeO_6_, where M = Cu, Ni, Zn, or Mg, Na_2_Zn_2_TeO_6_ demonstrated excellent ionic conductivity owing to the largest interlayer space (∼5 Å), which provides a large Na^+^ ion migration path as compared to the other analogues.^36^ Considerable attention has been paid to further increasing this interlayer space via the aliovalent substitution of Ga and Ca in the Na_2_Zn_2_TeO_6_ framework.^40−42^ This type of substitution triggers the ion transport in two ways: (1) increasing the charge carrier density and (2) enlarging the ionic migration pathway in Na_2_Zn_2_TeO_6_.

### Ionic Conduction Mechanism of Sulfide-Based Electrolytes

Sodium superionic conductors mainly refer to the sodium thiophosphates, and considerable attention has been paid to AS^3^B applications owing to their excellent ionic conductivity and high ductility. A class of lithium analogue sulfides such as Na_3_PS_4_, Na_11_Sn_2_P_2_S_12_, Na_3_SbS_4_, etc. have emerged as excellent ionic conductors.^43^ The high σ is devoted to the high availability of vacant sites in the crystal framework contributing to the hopping of ions. This prerequisite for high ionic conduction varies from structure to structure. Generally, Na_3_PnS_4_ (where P = Sb, Sn) crystallizes in two forms (i.e., cubic (space group *I*43*m*) or tetragonal (space group *P*421*c*)) as shown in Figure 2d.^44^ Primarily, cubic Na_3_PnS_4_ contains a body-centered cubic (BCC) arrangement of PnS_4_^3–^ units, whereas the tetragonal Na_3_PnS_4_ shows a rotation of PnS_4_^3–^ around the [111] axis that goes along with a minor tetragonal distortion along the *c* axis. This results in the availability of one and two Na^+^ ions in the cubic and tetragonal polymorphs of Na_3_PnS_4_, respectively. The ion migration pathway of fully occupied Na^+^ is shown in Figure 2d. In the case of full occupation, the Na^+^ transport properties completely depend on the concentration defect, synthesis parameters, and crystal structure.

Similarly, as derived from Li_10_GeP_2_S_12_, Na_10_Sn_2_P_2_S_12_ is reported to be an excellent sodium-ion conductor for AS^3^B. It crystallizes into the tetragonal phase (space group, *I*4_1_/*acd*) that features a peculiar three-dimensional chessboard-like framework of SnS_4_ and PS_4_ tetrahedra that expands the Na^+^ ion channels along the *c* axis and within the ab planes, as well depicted in Figure 2e. Ab initio molecular dynamics (AIMD) simulation for Na_11_Sn_2_P_2_S_12_ showed that the Na^+^ ions occupy only octahedral sites, and ionic conduction is solely three-dimensional in nature.

## Inorganic Electrolytes

Inorganic electrolytes (IEs) are the crucial component of AS^3^B like the electrode materials. The selection of suitable IE solely determines the practical characteristics of batteries such as power and energy density, ultralong stability, and safety standards. For practical AS^3^B, an ideal electrolyte must inherit the following traits:

- IEs should have excellent ionic conductivity of ∼10^–2^ S cm^–1^ at different operating temperatures (ambient to moderate).
- IEs should have negligible electronic conductivity.
- IEs should be thermally stable.
- IEs should have a wide electrochemical potential stability window of ≥5 V vs Na^+^/Na (to increase the cell voltage and to eliminate the unwanted interfacial reactions with high-voltage cathodes and Na metal anodes).
- IEs should be chemically inert toward the Na metal anode and Na-containing cathode.
- IEs should be dense and mechanically strong, capable of suppressing dendrite formation.
- IEs should be easily processable into thin film.
- IE preparation should be cost-effective, environmentally friendly, and easily scaled up for commercial production.

**Figure 3:**
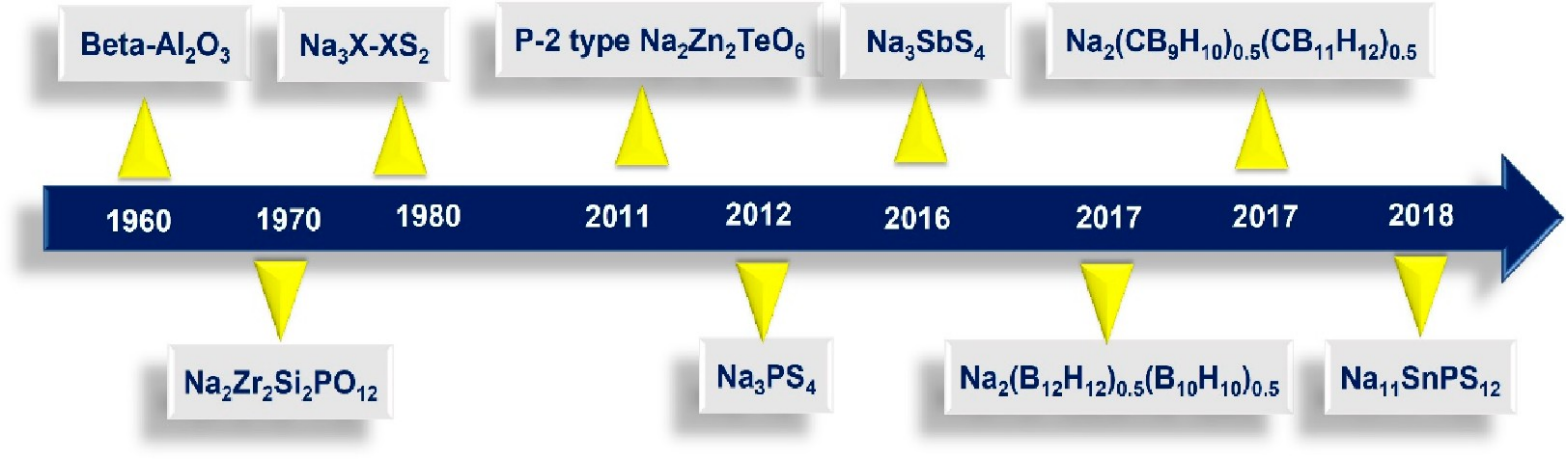
Hierarchical development of inorganic electrolytes for AS^3^B.

In the context AS^3^B, various type of IEs such as β-alumina, NASICONs, sulfides, borohydrides, and antiperovskites have been reported and are depicted in Figure 3.

### β-Alumina Electrolytes

The Na-β electrolytes were initially reported by Yao and Kummer’s group in 1967, realizing the practical sodium–sulfur (Na–S) batteries. The typical configuration of a Na–S battery consists of a Na anode, a β′′-Al_2_O_3_ electrolyte, and a S cathode. Such early reported β-alumina electrolytes are highly investigated for elevated temperature while operating rechargeable sodium–sulfur batteries. β-Alumina electrolytes exist in two types of distinguishable crystal structures based on the block layering sequences and respective chemical compositions [i.e., Na-β-Al_2_O_3_ with the chemical formula of Na_2_O·8–11Al_2_O_3_; space group *P*6_3_/*mmc* (hexagonal, a_0_ = 0.559 nm, c_0_ = 2.261 nm) and Na-β′′-Al_2_O_3_ with the chemical formula of Na_2_O·5–7Al_2_O_3_ and space group: *R*3̅*m* (rhombohedral, a_0_ = 0.560 nm, c_0_ = 3.39 nm)].^31,48^ Both types of crystal structures accommodate excess sodium as compared to the ideal structure of Na_1+*x*_Al_11_O_17+*x*/2_ (0.15 < *x* < 0.3), where the concentration of sodium ions is fulfilled by oxygen ions and exhibits an anisotropic Na-ion conductivity. The typical crystal structures of Na-β-Al_2_O_3_ and Na-β′′-Al_2_O_3_ are responsible for the ion-transport mechanism. It is evident that Na-β-alumina with a hexagonal structure consists of two spinel blocks and a single conduction plane, where Na-β′′-Al_2_O_3_ consists of one conducting plane interstitially stacked among three spinel blocks. Mobile sodium ions reside in these conducting planes. Na-β′′-Al_2_O_3_ also inherits a higher concentration of sodium ions than Na-β-Al_2_O_3_. Among both electrolytes, the highest σ of 0.2–0.4 S cm^–1^ at 300 °C is demonstrated by the single crystal of Na-β′′-Al_2_O_3_, which is higher than the polycrystalline phase (0.24 S cm^–1^ at 300 °C). Despite a high σ, further progress is retarded by the complicated process and requirement of high sintering temperature (i.e., 1200–1500 °C). The pure Na-β′′-Al_2_O_3_ phase is difficult to achieve due to its thermodynamic instability and its easy decomposition to an Al_2_O_3_ and Na-β-Al_2_O_3_ mixture at an elevated temperature of about 1500 °C. Consequently, the obtained product contains a mixture of Na-β-Al_2_O_3_ and Na-β′′-Al_2_O_3_.

To further enhance the conductivity of Na^+^ in Na-β′′-Al_2_O_3_, several dopants are used to stabilize the dominant highly ion-conductive Na-β′′-Al_2_O_3_ phase. Accommodating excess Na^+^ ions in interstitial sites also results in the formation of the Na-β′′-Al_2_O_3_ phase. Several monovalent (Li^+^), divalent (Cd^2+^, Co^2+^, Cu^2+^, Mg^2+^, Mn^2+^, Ni^2+^, and Zn^2+^), and tetravalent (Ti^4+^) cations, pentavalent (Nb^5+^) or metal oxides Cr_2_O_3_ (0.15 wt %), 3YSZ (5 vol %), 3YSZ (5 wt %), 8YSZ (15 wt %), TiO_2_ (1.5 wt %), SnO_2_ (1 mol %), CoO (1 wt %), MnO_2_ (0.5 wt %, 1 wt %), Ta_2_O_5_ (0.3 wt %), and Na_2_O(25 wt %)) with optimal concentration are doped into the crystal framework to stabilize the Na-β′′-Al_2_O_3_ phase.^49,50^

Additionally, replacing Al^3+^ in the spinel blocks by Mg^2+^ having a lower charge led to the imbalance in charge and gradient change of mobile Na^+^ in the conduction plane responsible for the ionic conduction. MnO_2_ (1 wt %) containing sodium β-alumina sintered at 1600 °C exhibited a maximum fraction of a 98% Na-β′′-Al_2_O_3_ phase. The doping also enables a lower sintering temperature as well as densification and fracture strength of the electrolyte.

### NASICON-Type Electrolytes

**Figure 4:**
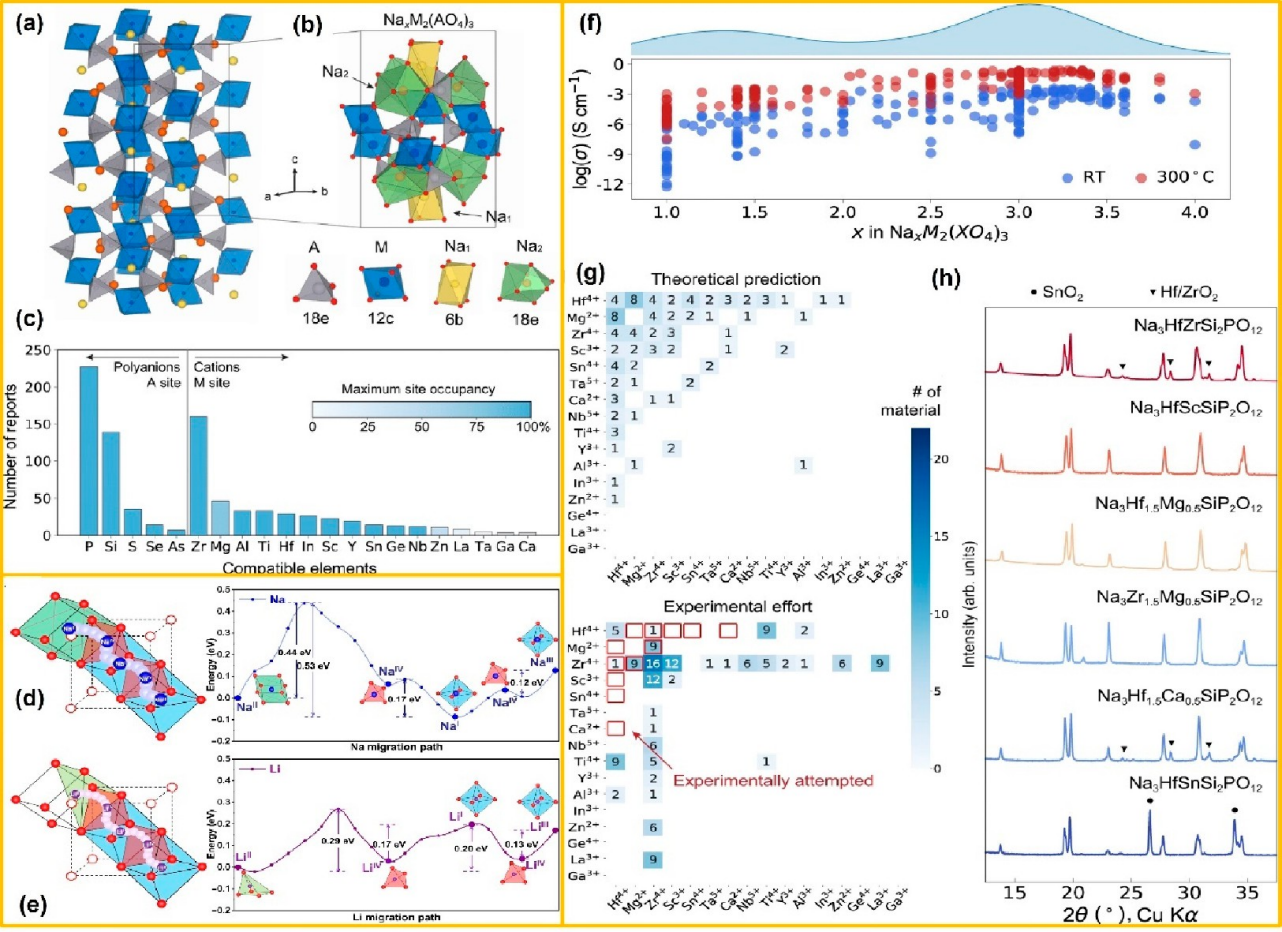
(a) Rhombohedral crystal structure of NASICON (Na_4_M_2_(AO_4_)_3_). (b) Local structure exhibiting characteristic cation sites in the NASICON framework. (c) Literature report regarding NASICONs having a specific element. (f) Experimentally determined ionic conductivity plot log scale of the *y*-axis vs *x*-axis Na content of NASICONs at ambient temperature and 300 °C reported based on the literature. (g) Distribution curve between theoretically (DFT) predicted Na-rich ground states/likely synthesized-NASICONs and experimentally synthesized Na-rich NASICONs (bottom panel). (h) XRD patterns of experimentally synthesized NASICONs with impurities (Hf/Zr)O_2_) and SnO_2_, respectively. Panels a–c and f–h are reproduced from ref (54). Available under a CC-BY 4.0 license. Copyright 2021 Ouyang, B., et al. (d) Energy barriers for single-ion migration of Na^+^ (blue color) following the Na_II_-Na_IV_-Na_I_-Na_IV_-Na_III_ trajectory. (e) Energy barriers for Li^+^ (purple color) having a Li_II_-Li_IV_-Li_I_-Li_IV_-Li_III_ trajectory path. Panels d and e are reproduced from ref (56). Available under a CC-BY 4.0 license. Copyright 2022 Zhu, L., et al.

In 1976, Hong and Goodenough introduced NASICON (a Na super ionic conductor) with the chemical formula Na_1+*x*_Zr_2_Si_*x*_P_3_O_12_ (0 ≤ *x* ≤ 3) derived from parent compound NaZr_2_P_3_O_12_ with the partial replacement of P^5+^ sites by Si^4+^. The Na_1+*x*_Zr_2_Si_*x*_P_3_O_12_ electrolyte with *x* = 2, Na_3_Zr_2_Si_2_PO_12_exhibited the highest σ (6.7 × 10^–4^ S cm^–1^ @ 25 °C and 2 × 10^–1^ S cm^–1^ @ 300 °C). Na_1+*x*_Zr_2_Si_*x*_P_3_O_12_ (1.8 ≤ *x* ≤ 2.2) generally crystallizes into polymorphs (i.e., monoclinic (space group *C*2/*c*) and rhombohedral (space group, *R*3*c*) crystal structures), as shown in Figure 4(a and b). The two phases comprise SiO_4_ tetrahedra, PO_4_ tetrahedra, and ZrO_6_ octahedra positioned at the corners, creating a “hexagonal bottleneck” for Na^+^ migration and having the shortest diameter of 4.6 Å with the anion framework consisting of four different sites for cations.^51^ The large space in the crystal framework facilitates abrupt Na^+^ migration through the bottleneck, thus increasing the σ. In the rhombohedral crystal structure, sodium resides in two different sites (i.e., Na1 and Na2), forming a three-dimensional diffusion network, whereas in the distorted monoclinic phase, original Na2 splits into Na2 and Na3 sites to form two distinct migration pathways (i.e., Na1–Na2 and Na1–Na3, respectively). The Na2 site is capable of accommodating 3 mol of Na^+^ in the rhombohedral phase space.^52^

The substitution of a low-valence ion in the NASICON crystal framework modifies the composition by introducing more Na^+^ into the lattices, causing an increased density of mobile sodium ions in accordance with the charge balance principle. Thus, a significant improvement in σ is observed for substituting the Na site (Li^+^, K^+^), Zr/Si site (Cu^2+^, Mn^2+^, Ca^2+^, Zn^2+^, Al^3+^, Mg^2+^, Gd^3+^, La^3+^, Nd^3+^, Sc^3+^, Er^3+^, Fe^3+^, Tb^3+^, Eu^3+^, Y^3+^, Yb^3+^, Ce^4+^, Ti^4+^, Si^4+^, etc.), and P site (Si^4+^, As^5+^, and S^6+^). Moreover, the substitution of similar radii atoms with the parent atoms effectively widens the size of bottlenecks and lowers the activation energy for ion transport, which in turn increases the σ.

Further enhancement in σ can be achieved by improving the packing density, optimizing the secondary phase, and modifying the grain boundary.^53^ Recently, Ceder et al. theoretically predicted the stability rules of NASICON based on the materials discovery, physical interpretation, and machine-learning tools.^54^ Taking into account the crystal structure of NASICON and respective Wyckoff positions for cations as shown in Figure 4(a and b), they performed phase diagram calculation analysis to determine the energy of NASICONs in the chemical space of Na-M_1_-M_2_-A-B-O. They screened over 21 metals and determined 3881 distinct compositions. It is anticipated from previous reports that the Na content (*x*) in the NASICON framework varies from 0 to 4, and there is a wide range for choosing the cations to coexist at the M and A sites in the NASICON framework, as depicted in Figure 4c. Indeed, multiple parameters play a role in limiting the phase stability, such as bond compatibility across different site conditions and the propensity for an element to use a specific interaction condition. Furthermore, the Na^+^ migration barriers for NZSP were estimated to determine its coordinated structure in the dilute limit of single Na^+^ without any other cations. The Na^+^ migration barrier is found to be 0.44 eV, which is lower than the Li^+^ migration barrier (0.29 eV) as shown in the relative energy graph in Figure 4(d and e). They experimentally validate the reported σ versus the Na content (*x*) for nearly 200 NASICON compositions, as shown in Figure 4f. It is clear that σ increases with the increase in Na content in the Na_*X*_M_2_(XO_4_)_3_ for up to *X* = 3, which tends to be the optimum Na-rich stochiometric composition.

Thermodynamic stability studies reveals that NASICONs containg metals such as Hf^4+^, Zr^4+^, Ta^5+^, and Sc^3+^ exhibit higher stability and compatibility than other metals. However, a stabilized NASICON crystal structure is difficult to achieve in NASICON containing Ca^2+^, Zn^2+^, Ge^4+^, and La^3+^ in the crystal framework. NASICONs with a low Na content exhibit a higher stability. They categorized the NASICONs into three types (i.e., thermodynamic ground states (GS), likely synthesizable (LS), and unlikely synthesizable (US)). The initial two types of NASICONs having an M–M′ pair with a Na content of *x* ≥ 3 are shown in Figure 4g. The bottom panel displays a similar prediction as witnessed by the experimentally reported NASICONs. The study revealed that there are nearly 60 unexplored compositions feasible for the GS/LS NASICON synthesis. They validated their theoretical prediction by synthesizing six NASICON samples, as shown in Figure 4h. Out of these six prepared samples, one sample with a chemical composition of Na_3_HfSn(SiO_4_)_2_(PO_4_) shows the large fraction of the SnO_2_ impurity phase. Recently, Yao et al. developed a 6 V electrochemical stability window using Na_3.4_Mg_0.1_Zr_1.9_Si_2.2_P_0.8_O_12_ with a superior σ of 3.6 mS cm^–1^, which confirms the larger migration of Na^+^ compared to that of the facile Na_3_Zr_2_Si_2_PO_12_. The introduction of the Mg^2+^ site at the Zr^4+^ site effectively resulted in a higher stripping/plating behavior over 2000 h at 0.1 mA cm^–2^ without any short-circuiting.^55^

### Sulfide-Based Electrolytes

**Figure 5:**
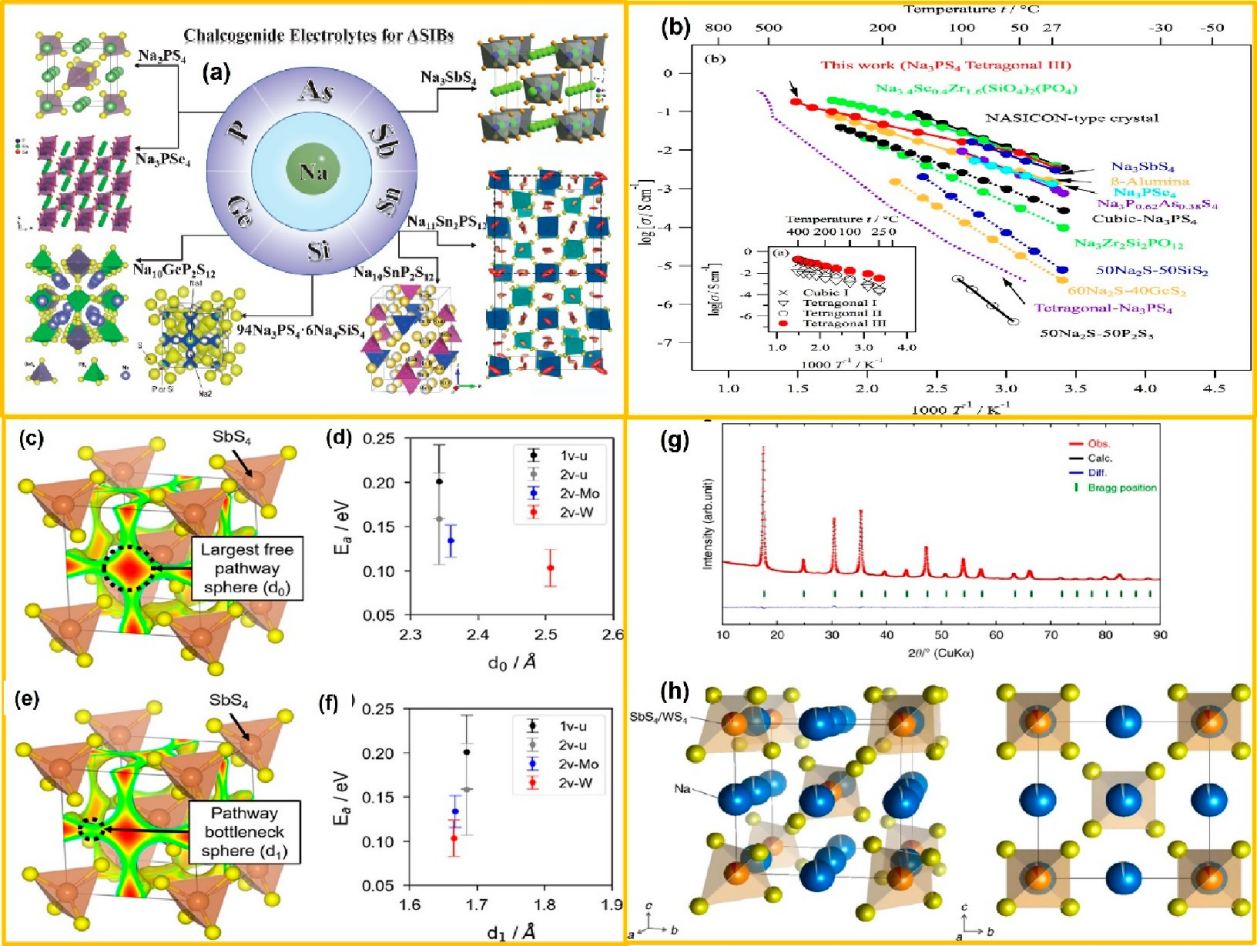
(a) Various chalcogenide-based electrolytes reported for AS^3^B. Panel a is reproduced from ref (58). Copyright 2020 Editorial Board of Acta Physico-Chimica Sinica. (b) Reported ionic conductivities of existing IEs for AS^3^B. Panel b is reproduced from ref (59). Copyright 2018 Elsevier Ltd. Schematic illustrations of the (c) Na Wyckoff site cage size and (d) DFT-MD Na^+^ ion activation energy (*E*_a_) with error bars vs d0 plot. (e) Pathway bottleneck extracted from DFT-optimized structures. (f) DFT-MD *E*_a_ with error bars vs d_1_ plot. Panels c–f are reproduced from ref (60). Copyright 2020 American Chemical Society. (g) Rietveld refinement of X-ray powder diffraction data and (h) crystal structure of cubic Na_2.88_Sb_0.88_W_0.12_S_4_. Panels g and h are reproduced from ref (61). Available under a CC-BY 4.0 license. Copyright 2019 Hayashi et al.

Sulfide electrolytes are the prevailing electrolytes for IEs in the AS^3^B and are highly preferred over their oxide’s analogue. Owing to the advantage of excellent ionic conductivity, low synthesis temperature, superior mechanical properties, isotropic ionic conductions with negligible grain boundary resistances, reduced production cost, and an intimate contact electrode–electrolyte interface, sulfide-based oxides served as reliable electrolyte materials.^57^ Various types of the reported chalcogenide-based SSEs with corresponding σ are shown in Figure 5(a and b). Hyashi et al. first reported the Na_3_PS_4_-based glass–ceramic electrolyte in two crystal forms: cubic (space group, *P*421*c*; a_0_ = b_0_= 0.69520 nm, c_0_ = 0.70757 nm) and tetragonal phases of 75Na_2_S-25P_2_S_5_ glass at 270 and 400 °C, corresponding to the cubic (*P*421*c*) and tetragonal phases (space group *I*43*m*; a_0_ = b_0_ = c_0_ = 0.70699 nm), respectively. As an optimized superionic cubic Na_3_PS_4_ crystal with the composition of 75Na_2_S-25P_2_S_5_, it exhibited a σ of 2.0 × 10^–4^ S cm^–1^ with a potential window of about ∼5 V and an appreciable electrochemical stability against Na. Generally, sulfide-based electrolytes are categorized into glass and glass-ceramic electrolytes.

Both the cubic and tetragonal phases of NaPS_4_ have minute differences with less than a 0.2% volume change in both phases. In the cubic polymorph, the Na resides at the Na1(6b) sites, whereas in the tetragonal phase, the Na1 sites split into Na1(2a) and Na2 (4d) sites are shown in Figure 5(c and d). The cubic phase shows a higher σ value when compared to the tetragonal phase.

Sulfur is highly efficient in providing a wide ion-transport pathway for rapid sodium ion migration due to its negligible electronegativity with weakly bonded ions and a larger radius. The aliovalent doping with Si, Se, Sn, Ge, and S^2–^ in P sites generates vacancies in the Na_3_PS_4_ crystal framework, significantly providing fast sodium ion migration and thus resulting in enhanced ionic conductivity. Despite its superior σ, sulfide-based electrolytes are prone to instability toward air (or moisture sensitivity). These sulfide electrolytes undergo hydrolysis when encountering moisture and thus releases noxious H_2_S gas. It is theoretically proven that Na vacancies are induced upon the doping of cations in Na_3_SbS_4_. The cation doping plays a vital role in improving σ, while the diffusion is further characterized in terms of the concerted motion of sodium ions.

Recently, Jiao et al. synthesized cubic-phase Mn-substituted Na_3_SbS_4_ attributed to the low energy barrier compared to that of Na_3_SbS_4_, resulting in the high σ of 2.05 mS cm^–1^ with a wide electrochemical window of 5 V. Na_3.24_Mn_0.08_-Sb_0.92_S_4_ with an increased crystallinity effectively inhibits the sodium dendrite growth and excellent air stability.^62^ Similarly, Tateyama et al. investigated the ionic conduction mechanism of W- and Mo-doped Na_3_SbS_4_ and the role of aliovalent cation substitution in conductivity improvement using density functional theory molecular dynamics (DFT-MD) calculations. The comparative results of W- and Mo-doped Na_3_SbS_4_ revealed that the improved conductivity is accredited to the decrease in Na^+^ activation energy. This parameter is accredited to the broadening of Na Wyckoff site cages induced by the smaller WS_4_/MoS_4_ tetrahedral volume relative to the SbS_4_ volume as shown in Figure 5(c–f).

Comparatively, WS_4_/MoS_4_ displays a minimal volume with an increased bond angle deviation (5.56 Å, ±0.22°) compared to that of SbS_4_ (6.82 Å, 0°). Similarly, the lattice parameter calculated from the DFT tends to be 7.244 Å for MoS_4_ and 7.248 Å for WS_4_. Hyashi et al. also reported a highly ion-conductive Na_2.88_Sb_0.88_W_0.12_S_4_, which is the same as Li_10_GeP_2_S_12_. An unprecedented σ of 32 mS cm^–1^ via partially doping Sb in Na_3_SbS_4_ with tungsten is anticipated for the induced Na vacancies and the transition from the tetragonal phase to the cubic phase as shown in Figure 5(g and h). This sulfide-based inorganic solid electrolyte delivers an extensive tolerance of H_2_S gas under ambient conditions, which is very helpful in improving the safety and reducing manufacturing costs.

### Borohydride-Based Electrolytes

**Figure 6:**
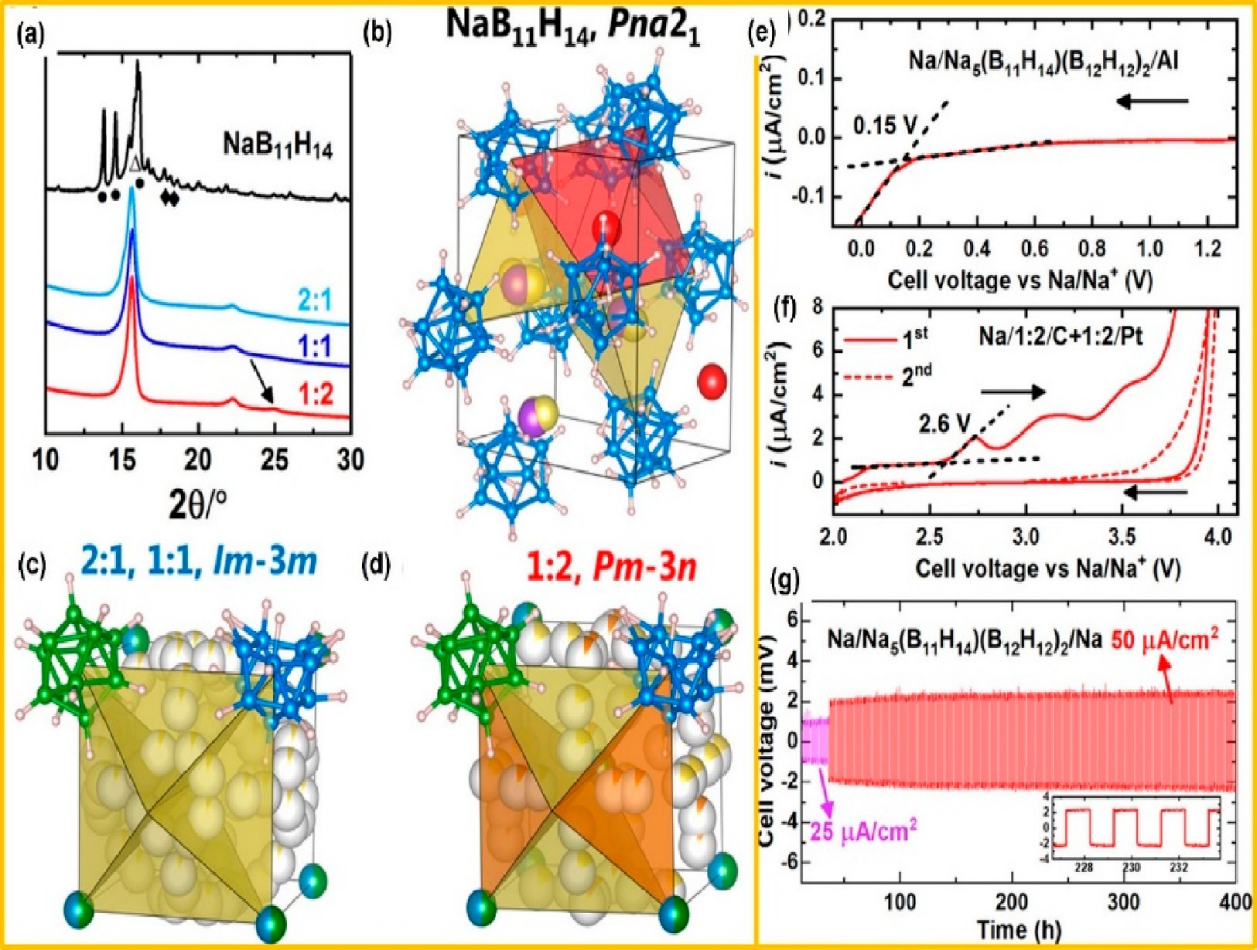
(a) X-ray diffraction pattern of NaB_11_H_14_ and Na_*x*+2*y*_(B_11_H_14_)_*x*_(B_12_H_12_)_*y*_. (b–d) Chemical structures of NaB_11_H_14_ and Na_*x*+2*y*_(B_11_H_14_)_*x*_(B_12_H_12_)_*y*_. LSV curves of (e) the Na/Na_5_(B_11_H_14_)(B_12_H_12_)_2_/Al cell in the range of 1.3 to −0.1 V vs Na/Na^+^ and (f) the Na/Na_5_(B_11_H_14_)(B_12_H_12_)_2_/Na_5_(B_11_H_14_)(B_12_H_12_)_2+C_/Pt cell from 2.0 to 4.0 V vs Na/Na^+^ at a scan rate of 0.05 mV/s at 60 °C. (g) GCD characteristics of a symmetric Na/Na_5_(B_11_H_14_)(B_12_H_12_)_2_/Na cell at 60 °C with current densities of 25 μA cm^–2^ for the initial 24 h and 50 μA/cm^2^ for the rest of the measurements (1 h for each direction). Figure 6 is reproduced from ref (64). Copyright 2020 American Chemical Society.

Recently, complex-based electrolytes have emerged as a promising electrolyte for AS^3^B. Unlike Na-β-alumina- and NASICON-based electrolytes, borohydrides inherit the cage-like symmetrical quasi-spherical architectures. Different hydrides anion such as [BH_4_]^−^, [NH_2_]^−^, [AlH_4_]^−^, and [AlH_6_]^3–^ form complexes with different alkali ions that exhibit unique applications.^63^ Rehmof et al. introduced NaB_11_H_14_ nido-borates as a new building block for the solid electrolytes by employing an economically favorable low-temperature method with NaBH_4_ as a chemical feedstock. The as-prepared NaB_11_H_14_ nido-borates with closo Na_2_B_12_H_12_ exhibited an excellent σ of 4 mS cm^–1^ @20 °C.^64^ The characteristic crystal structure was explored using a two-dimensional magnetic resonance techniques. The XRD pattern and crystal structure of pristine NaB_11_H_14_ and combined Na_*x*+2*y*_(B_11_H_14_)_*x*_(B_12_H_12_)_*y*_ (*x*:*y* ratios of 2:1, 1:1, and 1:2) are shown in Figure 6(a–d). The space group obtained for 2:1 and 1:1 is *Im*3*m*, whereas for 1:2 it is *Pm*3*n*. Furthermore, the Na^+^ per unit formula is 1.67 for the 1:2 ratio, which is greater than the other two ratios (2:1–1.33 and 1:1–1.5). Overall, the 1:2 ratio of Na_5_(B_11_H_14_)(B_12_H_12_)_2_ has the highest σ (4 mS cm^–1^). The optimized Na/Na_5_(B_11_H_14_)(B_12_H_12_)_2_/Al and Na/Na_5_(B_11_H_14_)(B_12_H_12_)_2_/Pt cells exhibited an open-circuit voltage of ∼2 to 4 V vs Na/Na^+^ (Figure 6e). It displays a wide electrochemical stability window of ∼3.5 V, indicating the strong oxidative current as shown in Figure 6f. The as-fabricated Na/Na_5_(B_11_H_14_)(B_12_H_12_)_2_/Na delivered an appreciable cycling stability for 400 h at 50 μA cm^–2^, switching the current direction every 1 h as shown in Figure 6g. Recently, Guo et al. developed a high-voltage hydroborate-based Na_3_B_24_H_23_-5Na_2_B_12_H_12_ solid electrolyte battery of 4 V that features a high σ of 1.42 mS cm^–1^, a high transference number of 0.97, and a 6 V wider electrochemical stability window, which is known to be higher than that of the other IEs for AS^3^B.^65^

### Antiperovskite-Based Electrolytes

Antiperovskite-based electrolytes such as NaMgF_3_, (K,Na)MgF_3_, Na_2.9_Sr_0.05_OBr_0.6_I_0.4_, Na_9_Al(MoO_4_)_6_, Na_3_OBr, and Na_4_OI_2_ are newly emerged inorganic solid electrolytes for AS^3^B with an improved σ.^66^ A widely investigated Na_3_OBr exhibits a cubic antiperovskite-type chemical structure (space group *Pm*3*m*, a = 4.5674(1) Å). In this structure, corner-sharing ONa_6_ octahedra with Br^–^ ions are located on the A site of the crystal framework. On the other side, Na_4_OI_2_ exhibits a tetragonal antiperovskite crystal structure (space group *I*4/*mmm*, a = 4.6729(1) Å, and c = 15.9556(5) Å). Based on the theoretical calculation, both Na_3_OBr and Na_4_OI_2_ show low activation energies for the characteristic Na^+^-ion migration pathways.^67^

### P-2-Type Layered Oxide-Type Electrolytes

**Figure 7:**
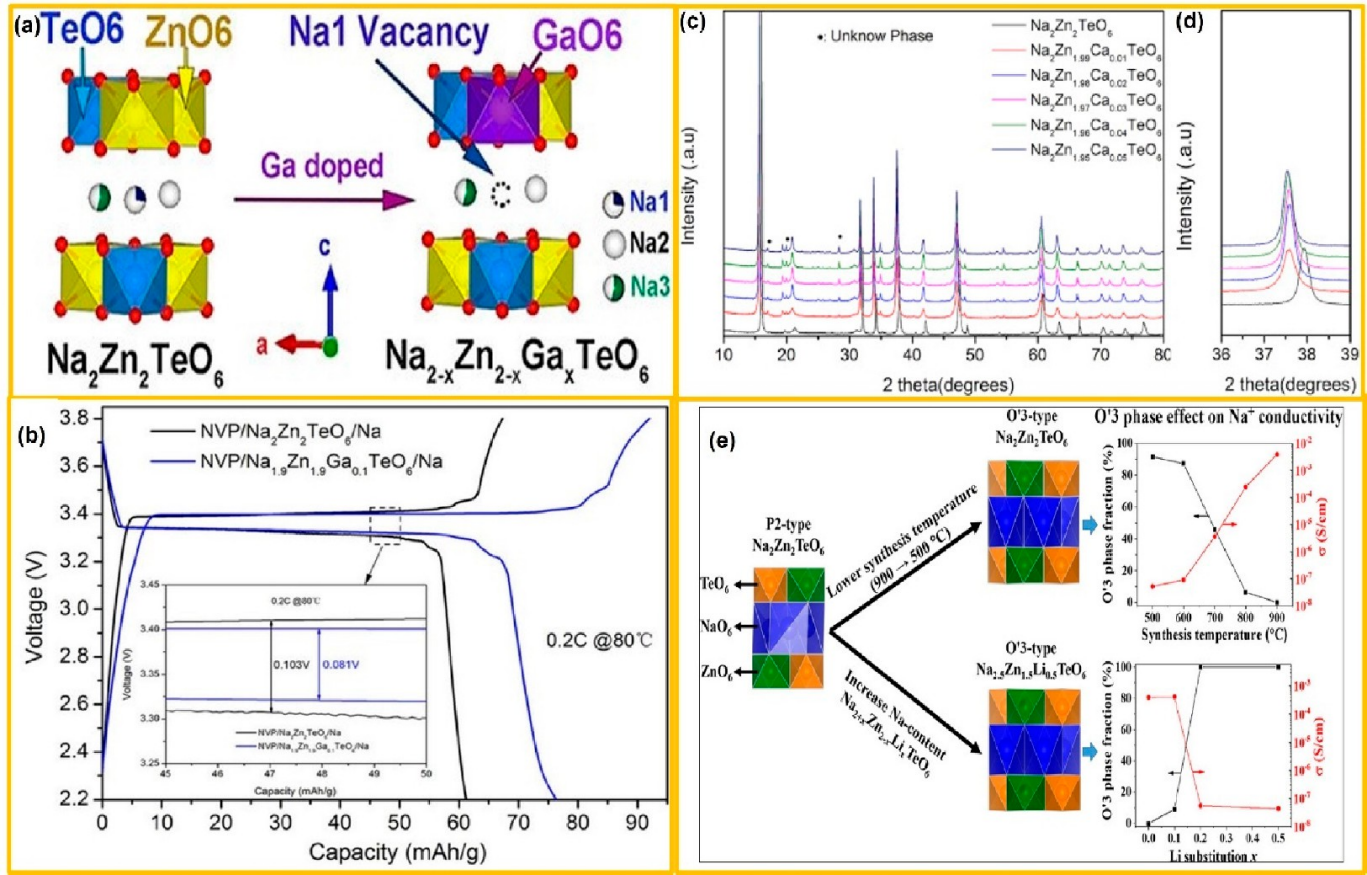
(a) Crystal structure of Na_2–*x*_Zn_2–*x*_Ga_*x*_TeO_6_. (b) Charge/discharge characteristics of the cell with configuration NVP/NZTO-Gx/Na (*x* = 0, 0.1) with 0.2 C at 80 °C. Panels a and b are reproduced from ref (41). Copyright 2018 Wiley VCH Gmbh. (c) X-ray diffraction pattern of NZTO-C*x* (*x* = 0–0.05) samples. (d) Enlarged view of the X-ray pattern. Panels c and d are reproduced from ref (42). Copyright 2018 Elsevier Ltd. (e) Crystal structures of P2-type and O′3-type Na_2_Zn_2_TeO_6_ with corresponding ionic conductivity values. Panel e is reproduced from ref (39). Copyright 2020 American Chemical Society.

In 2011, Evstigneeva et al. introduced a class of P-2-type layered Na-based compounds with the stoichiometric chemical formula of Na_2_M_2_TeO_6_ (M = Ni, Co, Zn, Mg). They first reported the crystal structure of Na_2_Ni_2_TeO_6_ and Na_2_Zn_2_TeO_6_, revealing that Na_2_M_2_TeO_6_ (M = Zn, Co, Mg) is constituted of MO_6/3_ and TeO_6/3_ octahedral units ordered in the plane along with shared edges.^36^ Interestingly, Na_2_Ni_2_TeO_6_ exhibited the space group *P*6_3_/*mcm* (a_0_ = 5.2074 Å, b_0_ = 11.1558 Å), and Na_2_Zn_2_TeO_6_ crystallizes into space group *P*6_3_22 (a_0_ = 5.2796 Å, b_0_ = 11.2941 Å). Furthermore, layered sodium-ion conductor Na_2_NiFeTeO_6_ reported the highest σ of 4 × 10^–3^ S cm^–1^ at 300 °C. The Na_2_Zn_2_TeO_6_ framework possesses a large two-dimensional reticulate Na^+^ migration interstitial pathway between two adjacent honeycomb structure layers, thus contributing to remarkable σ.^39^ Successive enhancement in σ was practiced via element doping (Mg, Ni, Ga, and Ca),^38,41,42^ aiming to achieve a higher Na^+^ concentration and enlarge the ionic transport pathway as shown in Figure 7a. Han et al. reported a gallium-doped P2-type layered material Na_1.9_Zn_1.9_Ga_0.1_TeO_6_ with an unprecedented σ of 1.1 × 10^–3^ S cm^–1^ at ambient temperature, surpassing the values achieved for NASICON and Na-β′′-Al_2_O_3_-based solid state electrolytes.

AS^3^B with a configuration of Na_3_V_2_(PO_4_)_3_/Na_1.9_Zn_1.9_Ga_0.1_TeO_6_/Na demonstrated excellent chemical and electrochemical performances by delivering a capacity of ∼70 mA h g^–1^ over 10 cycles with a current rate of 0.2 C at 80 °C as shown in Figure 7b.^68^ The exceptional σ was accounted for by the two reasons. The insertion of Ga^3+^ into the NZTO framework led to the enlarged Na^+^ ion migration pathway interlayers (∼5.58 Å) being more compared to NASICON and β/β″-alumina-based inorganic electrolytes. Increasing the Na-site vacancies upon the inclusion of Ga^3+^ in NZTO increased the carrier’s concentration and lowered the migration energy for Na^+^ ions. The interfacial resistance of Na_3_V_2_(PO_4_)_3_/Na_1.9_Zn_1.9_Ga_0.1_TeO_6_/Na was found to be lower than that of the Na_3_V_2_(PO_4_)_3_/Na_2_Zn_2_TeO_6_/Na (i.e., reduced from ∼360 to ∼225 Ω), revealing the interfacial improvement in the NZTO upon Ga^3+^ substitution.

**Figure 8:**
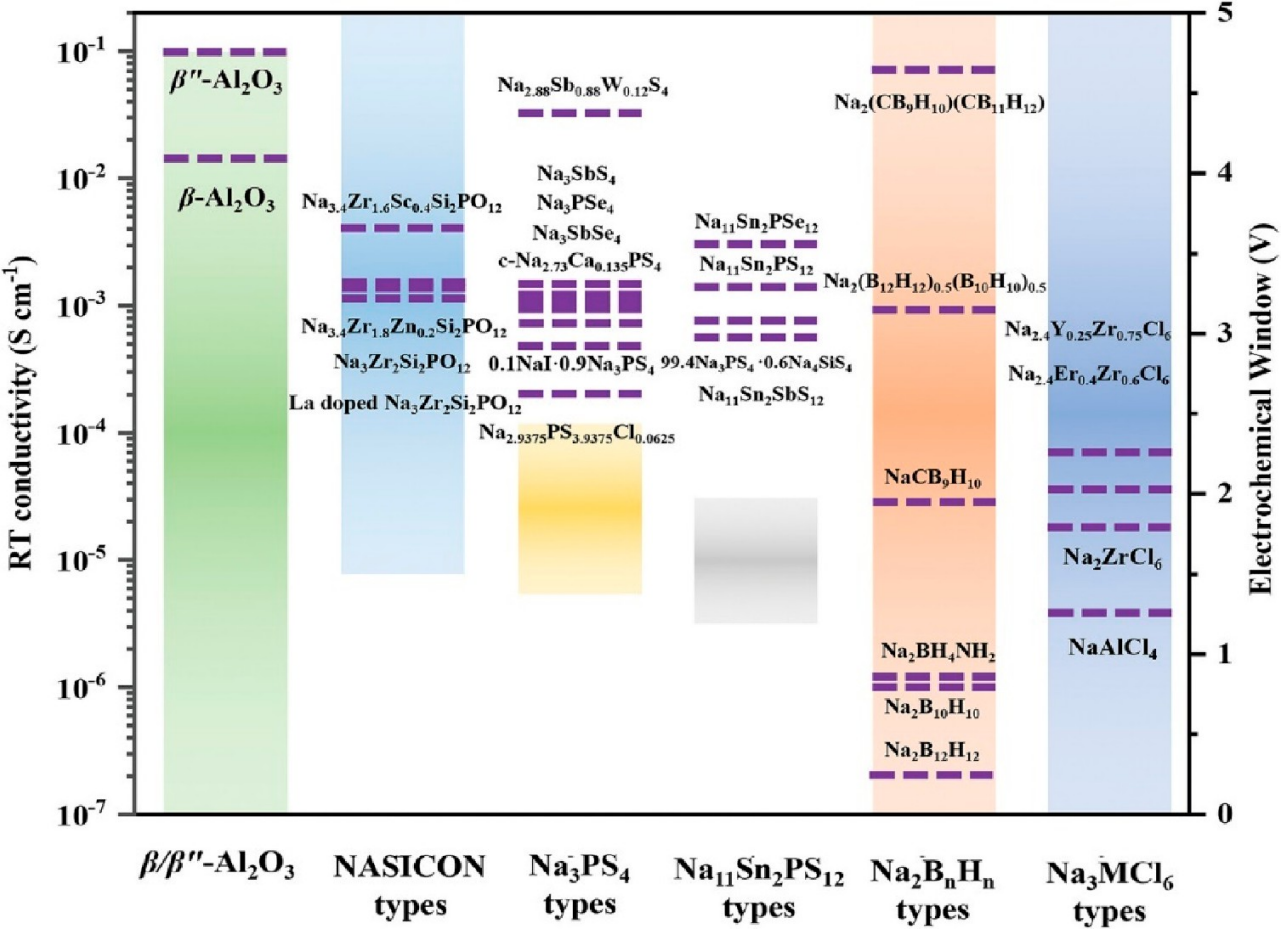
Ionic conductivity and electrochemical stability window of different IEs for AS^3^B. Figure 8 is reproduced from ref (29). Copyright 2023 Wiley VCH GmbH.

The same strategy for broadening the interlayer distance to achieve high σ was reported by Han et al. The group investigated the role of Ca^2+^ substitution in different concentrations in the P2-type layered NZTO (Na_2_Zn_2–*x*_Ca_*x*_TeO_6_ (*x* = 0–0.05)).^42^ Among all investigated samples, Na_2_Zn_2–*x*_Ca_*x*_TeO_6_ with *x* = 0.02 exhibited a maximum Na^+^ conductivity of 7.54  ×  10^–4^ S cm^–1^. This is anticipated to the grain-boundary modification and interlayer-interface elimination upon insertion of Ca^2+^. Additionally, Ca^2+^ substitution increases the interlayer spacing in NZTO as shown in Figure 7c. The peak shift over NZTO is due to the larger radius of Ca^2+^ (0.099 nm) compared to that of Zn^2+^(0.074 nm) as depicted in Figure 7d. Furthermore, the unknown phase observed in the XRD pattern of Na_2_Zn_2–*x*_Ca_*x*_TeO_6_ with *x* = 0.02 is anticipated to be the modification in the element concentration of the main phase but also densifies the Na_2_Zn_2–*x*_Ca_*x*_TeO_6_ with the *x* = 0.02 electrolyte, leading to a reduction in the interfacial resistance. Interestingly, Fjellvag et al. investigated the theoretical and experimental insights into the crystal structure and diffusion in the biphasic NZTO crystal framework.^39^ They reported first the existence of O′3-type phase for NZTO. Their investigation revealed that a pure P2-type phase can be attained for synthesis temperatures below 900 °C. For temperatures above 900 °C, mixed P2 and O′3 phases were formed. The pure O′3 phase is achieved only via the insertion of Li at the Zn site in the NZTO framework (Na_2+*x*_Zn_2–*x*_Li_*x*_TeO_6_, *x* = 0.2–0.5) as shown in Figure 7e. The synchrotron combined with the computational modeling indicates the balanced Na charge content after the incorporation of Li into the Zn. Based on computational modeling-enabled impedance spectroscopic studies, they stated that a reduction in the O′3-type phase solely contributed to the enhanced σ. Comparison analysis of inorganic electrolytes in terms of ionic conductivity (Table 1) and along with electrochemical stability windows of AS^3^B are shown in Figure 8.

**Table 1:** Ionic Conductivity Values of Inorganic Solid Electrolytes

Solid electrolyte	Ionic conductivity (S cm^–1^)	Activation energy (eV)	ref
**Na-β-Alumina**			
Single crystal Na-β-Al_2_O_3_	0.03 (25 °C)	0.13	(69)
	0.21 (300 °C)		
Polycrystalline Na-β-Al_2_O_3_	0.0012 (25 °C)	0.27 (25–200 °C)	(69)
	0.065 (300 °C)	0.15 (<200 °C)	
Single crystal Na-β′′-Al_2_O_3_	0.035 (25 °C)	0.33 (25–150 °C)	(50)
	1 (300 °C)		
Polycrystalline Na-β′′-Al_2_O_3_	0.36	0.18 (285–300 °C)	(70)
		0.16 (330–375 °C)	
α-Al_2_O_3_-doped 50 wt % YSZ	1.8 × 10^–2^ (350 °C)		(71)
Li-doped Na-β′′-Al_2_O_3_	5.6 × 10^–2^ (300 °C)	0.16 (300 °C)	(72)
Mg-doped Na-β′′-Al_2_O_3_	5.4 × 10^–2^ (300 °C)	0.19 (300 °C)	(73)
Mg-doped Na-β′′-Al_2_O_3_	1.8 × 10^–1^ (300 °C)	0.25 (350 °C)	(74)
Cr_2_O_3_-doped Na-β′′-Al_2_O_3_ (0.15 wt %)	8.2 × 10^–2^ (350 °C)	0.15(350 °C)	(75)
MnO_2_-doped Na-β′′-Al_2_O_3_ (1 wt %)	1 × 10^–1^ (350 °C)	0.21 (350 °C)	(76)
CoO-doped Na-β′′-Al_2_O_3_ (1 wt %)	6.1 × 10^–2^ (300 °C)	0.18 (300 °C)	(77)
SnO_2_-doped Na-β′′-Al_2_O_3_ (1 mol %)	5.2 × 10^–2^ (300 °C)	0.15 (300 °C)	(49)
**NASICONs**			
Na_1+*x*_Zr_2_P_3–*x*_Si_*x*_O_12_ (*x* = 2)	6.7 × 10^–4^ (25 °C)	0.29 (300 °C)	
	2.0 × 10^–1^ (300 °C)		
Na_3.2_Zr_1.9_Mg_0.1_Si_2_PO_12_	2.2 × 10^–3^ (27 °C)	0.27 (27 °C)	(78)
Na_3.4_Zr_1.8_Mg_0.2_Si_2_PO_12_	1.6 × 10^–3^ (25 °C)		(79)
Na_3.4_Zr_1.6_Sc_0.4_Si_2_PO_12_	4.0 × 10^–3^ (27 °C)	0.26 (27 °C)	(80)
Na_3_Zr_1.98_Nb_0.08_Si_2_PO_12_	2.1 × 10^–4^ (27 °C)	0.40 (27 °C)	(81)
Na_3_Zr_1.9_Ti_0.1_Si_2_PO_12_	3.8 × 10^–4^ (25 °C)	0.36 (25 °C)	(81)
Na_3_Zr_1.9_Yb_0.1_Si_2_PO_12_	1.7 × 10^–4^ (25 °C)	0.37 (25 °C)	(82)
Na_3.1_Zr_1.9_Nd_0.1_Si_2_PO_12_	6.9 × 10^–3^ (27 °C)		(83)
**Sulfides**			
Na_3_PS_4_	4.6 × 10^–4^ (RT)	0.20 (RT)	(84)
Na_3_PSe_4_	1.2 × 10^–3^ (RT)	0.21 (RT)	(85)
Na_3_SbS_4_	1.0 × 10^–3^ (RT)	0.22 (RT)	(86)
Na_10_SnP_2_S_12_	4.0 × 10^–4^ (RT)	0.36 (RT)	(47)
Na_11_Sn_2_PS_12_	1.4 × 10^–3^ (RT)	0.25 (RT)	(87)
**Borohydrides**			
Na_2_B_10_H_10_	<10^–6^ (RT)		(88)
Na_2_B_12_H_12_	<10^–6^ (RT)		(89)
Na_3_BH_4_B_12_H_12_	1 × 10^–3^ (RT)	0.34 (RT)	(90)
NaCB_9_H_10_	3 × 10^–2^ (RT)	0.20 (RT)	(91)
Na_2_(CB_9_H_10_)(CB_11_H_12_)	7 × 10^–2^ (RT)	0.22 (RT)	(92)
Na_2_(B_12_H_12_)_0.5_(B_10_H_10_)_0.5_	1 × 10^–3^ (RT)		(93)
**Antiperovskites**			
Na_3_OBr	2.7 × 10^–6^ (RT)	0.68 (RT)	(94)
Na_4_OI_2_	7.7 × 10^–5^ (RT)	0.64 (RT)	(94)
Na_2.9_Sr_0.05_OBr_0.6_I_0.4_	4.4 × 10^–4^ (RT)	0.62 (RT)	(95)
Na_9_Al (MoO_4_)_6_	8 × 10^–4^ (300 °C)	0.48 (300 °C)	(96)
**P-2-Type Layered Oxides**			
Na_2_Zn_2_TeO_6_	6.29 × 10^–4^ (RT)	0.327 (RT)	(41)
Na_2_Mg_2_TeO_6_	2.3 × 10^–4^ (RT)	0.231 (RT)	(40)
Na_2_Co_2_TeO_6_	(3.8–4.9) × 10^–4^ (25 °C)		(36)
Na_2_MgNiTeO_6_	2.13 × 10^–5^ (30 °C) Grain boundary	0.59 (30 °C)	(97)
Na_2_MgZnTeO_6_	0.90 × 10^–5^ (30 °C) Grain boundary	0.36(30 °C)	(97)
Na_2_LiFeTeO_6_	4 × 10^–3^ (300 °C)		(98)
Ga(0.1)-doped Na_2_Zn_2_TeO_6_	1.09 × 10^–3^ (RT)	0.271 (RT)	(41)
Ca(0.02)-doped Na_2_Zn_2_TeO_6_	7.54 × 10^–4^ (RT)	0.225 (RT)	(42)

## Electrode–Electrolyte Interface

**Figure 9:**
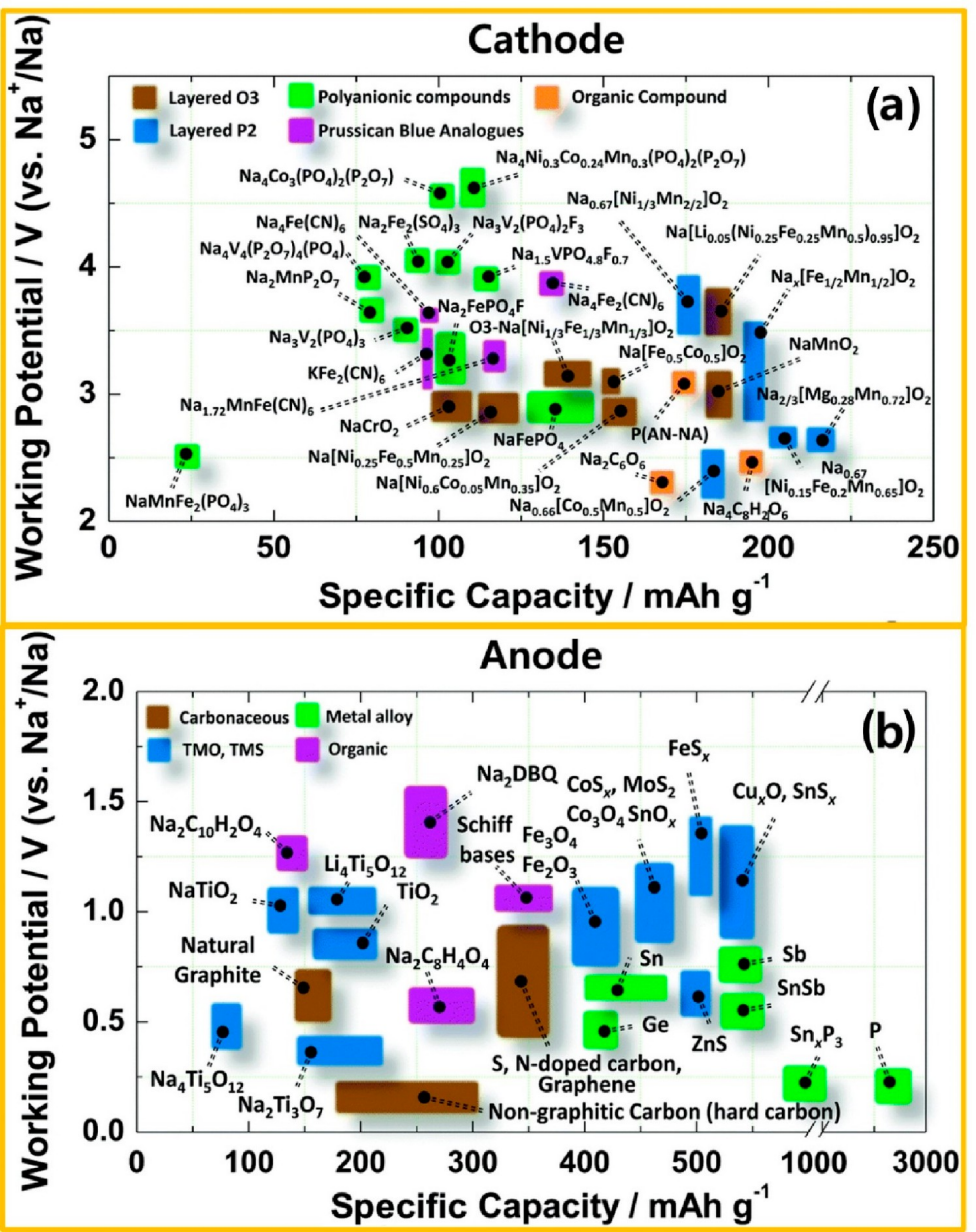
Recently reported electrode materials: (a) cathodes and (b) anodes for AS^3^B. Figure 9 is reproduced from ref (99). Copyright 2017 Royal Society of Chemistry.

The AS^3^B fabrication comprises a solid electrolyte along with a separator layer sandwiched between compressed electrode materials (cathodes/anodes). Electrode materials are commonly composed of active materials with some conducting agents. During operation, the Na ion diffused from active electrode material to the solid state electrolyte through the interface, resulting in electron migration from active material to current collector. Thus, the charge transfer through the interface in AS^3^B involves the cathode/electrolyte and anode/electrolyte interfaces. An ideal interface should possess compatibility, good mechanical strength, high ionic conductivity, and appreciable wetting characteristics. The current AS^3^B is prone to high interfacial resistance and poor interfacial contact due to an unintended volume change of electrodes during the Na^+^ intercalation/deintercalation, an inadequate contact area inheriting restricted ion transport pathways, and low active material loading.^100^ Owing to the high reactivity and large Na^+^ radius, the interfacial issues are more challenging in AS^3^B. The currently reported electrode materials (cathode/anode) are shown in Figure 9.

## Cathode–Electrolyte Interface

The solid–solid contact in the cathode–electrolyte interface is the most vital prerequisite for achieving high-performance AS^3^B. The hard texture of active materials and balanced ionic/electronic conductivity are responsible for the poor interfacial contact across the cathode–electrolyte interface.^101^ The strategies to improve the cathode–electrolyte interface are as follows:

### Composite Cathode Formation

The most common method for fabricating the interfacial contact is to develop a composite cathode by mixing active material and electrolyte, which has been widely used in both inorganic and organic solid electrolyte batteries. For example, Goodenough et al. proposed a NaTi_2_(PO_4_)_3_ composite cathode membrane of cross-linked poly(ethylene glycol) methyl ether acrylate (CPMEA), which served as a Na^+^ conducting binder with carbon black as the electron conductor.^102^ AS^3^B with the configuration of NaTi_2_(PO_4_)_3_, Na_3_Zr_2_Si_2_PO_12_/Na exhibited a discharge capacity of 110 mA h g^–1^ at a 0.2 C rate up to an initial 25 cycles and then 75 mA h g^–1^ at 1 C during the following 35 cycles. When the battery was tested at a C rate of 0.5 C, it delivered a discharge capacity of 94 mA h g^–1^. In all three tests, the Coulombic efficiency of 99.8 ± 0.2% was retained, indicating a high sodium deposition/stripping efficiency and appreciable electrochemical stability of AS^3^B. The same group has introduced AS^3^B with a plastic–crystal electrolyte interphase of succinonitrile and NaClO_4_ to improve the interfacial contact between the Na_3_Zr_2_(Si_2_PO_12_) pellet and the Na_3_V_2_(PO_4_)_3_ cathode.^103^ The presence of the plastic–crystal electrolytes in the cathode forms an intimate contact with the Na_3_V_2_(PO_4_)_3_ cathode to improve the efficiency of sodium-ion transfer. Similarly, a sodiated naflon-modified composite cathode was reported to improve the cathode (Na_3_V_2_(PO_4_)_3_/Na_3_Zr_2_Si_2_PO_12_/C, mass ratio 60:38:2)-Na_3_Zr_2_Si_2_PO_12_ electrolyte interface.^104^ The as-fabricated AS^3^B with a sodiated naflon-modified composite cathode exhibited a discharge capacity of 81.6 mA h g^–1^ at 20 mA g^–1^ and retained a high capacity of 62.23 mA h g^–1^ after 50 cycles. Unlike the liquid electrolyte, the introduced sodiated nafion in the swelling state facilitated a stable and rapid migration path for ionic conduction and served as a buffer layer for undesirable volume change in the cathode as a consequence of repeatedly charging and discharging.

**Figure 10:**
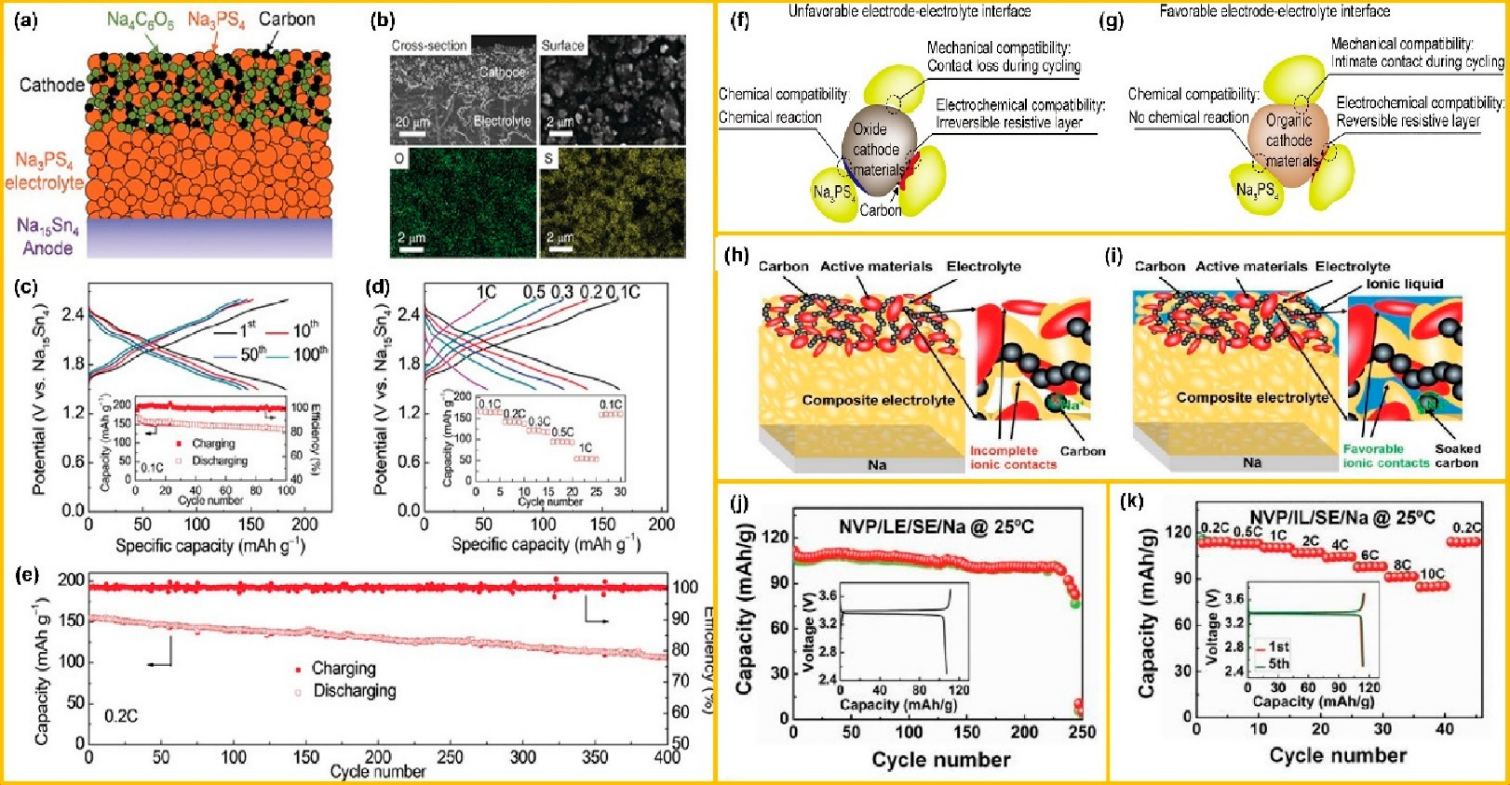
(a) Schematic diagram of AS^3^B with device configuration Na_4_C_6_O_6_/Na_3_PS_4_/Na_15_Sn_4_. (b) SEM image of catholyte cross-sectional interface and cathode surface with EDX mapping. (c) Charge–discharge characteristics of fabricated AS^3^B at various cycle numbers at 0.1 C at 60 °C. (d) Charge–discharge characteristics at different current rates. (e) Plot of capacity and Coulombic efficiency with respect to cycle number at 0.2 C at 60 °C. Panels a–e are reproduced from ref (111). Copyright 2018 Wiley-VCH GmbH. (f) Unfavorable interface between oxide cathode and Na_3_PS_4_. (g) Favorable electrode–electrolyte interface between the organic cathode and Na_3_PS_4_ electrolyte. Panels f and g are reproduced from ref (112). Copyright 2019 Elsevier Ltd. (h and i) Schematic diagram of interfaces (Na_3_V_2_(PO_4_)_3_/Na_3.3_Zr_1.7_La_0.3_Si_2_PO_12_/Na and Na_3_V_2_(PO_4_)_3_/ionic liquid/Na_3.3_Zr_1.7_La_0.3_Si_2_PO_12_/Na). (j and k) Cycling performance and Coulombic efficiency characteristics of cell Na_3_V_2_(PO_4_)_3_/Na_3.3_Zr_1.7_La_0.3_Si_2_PO_12_/Na and Na_3_V_2_(PO_4_)_3_/ionic liquid/Na_3.3_Zr_1.7_La_0.3_Si_2_PO_12_/Na at room temperature at 10 C for 10 000 cycles. Panels h–k are reproduced from ref (109). Copyright 2016 Wiley-VCH GmbH.

In another work, Yao et al. reported a composite cathode with a composition of Na_4_C_6_O_6_:Na_3_PS_4_:carbon 4:5:1 AS^3^B using Na_3_PS_4_ as an electrolyte as depicted in Figure 10a.^105^ The cold pressing technique promotes the direct contact between the cathode and electrolyte (Figure 10b), resulting in good battery performance. The assemble battery delivered a high specific capacity and high energy density of 184 mA h kg^–1^ and 395 W h kg^–1^, respectively. The cell shows an appreciable capacity retention of 76% for 100 cycles at 0.1 C and 70% for 400 cycles at 0.2 C, as shown in Figure 10(c–e). Furthermore, the postcycling studies also confirmed no new species formed during the battery cycling, resulting in excellent compatibility between Na_4_C_6_O_6_ and Na_3_PS_4_. The effort to address the cathode electrolyte interfacial concern was made by Yao et al., in which they proposed a composite cathode material with PTO-Na_3_PS_4_-C at a weight ratio of 20:(80–*x*):*x*, where *x* = 0.5, 10, 20, 28, and 33. Immediate contact of PTO with Na_3_PS_4_ helps the nanoparticles to bear the mechanical stress generated at the interface during the charge–discharge cycles. The as-fabricated AS^3^B delivered a specific capacity of 304 mA h g^–1^ at 0.1 C with an excellent capacity retention of 97% after 100 cycles.

Similarly, the composite cathodes are also reported for AS^3^B batteries with β-alumina electrolytes. For example, Honma et al. reported a Na_2_FeP_2_O_7_ glass ceramic cathode in which Na_2_FeP_2_O_7_ glass was successfully joined with a Na_2_OFe_2_O_3_P_2_O_5_ glass substrate by pressure less heat treatment cofiring at 550 °C.^106^ The composition of the cathode (i.e., Na_2_OFe_2_O_3_P_2_O_5_ glass, β″ alumina solid electrolyte, and acetylene black (AB)) was kept in the weight ratio of 72:25:3. The immediate contact at the cathode–electrolyte interface does not get peeled off during charging and discharging over a long period of 623 cycles.

### Surface Coating

Surface coating of active material particles with a thin layer of solid electrolytes is another effective technique to form intimate interfaces. For example, solid–liquid interfaces can be achieved by heating the glass electrolytes to around the glass-transition temperature, followed by favorable contacts after cooling to ambient temperature. Wang et al. proposed a substantial solution by introducing a thin layer of Na_3_PS_4_ coated on Mo_6_S_8_ using a solution method to achieve intimate contact at the Na_3_PS_4_–Mo_6_S_8_ interface as shown in Figure 10(f and g).^107^ The Na_3_PS_4_-coated Mo_6_S_8_ delivers a higher first cycle revisible capacity and capacity retention than does the bare Mo_6_S_8_ cathode. The as-fabricated cell containing an Na_3_PS_4_-coated Mo_6_S_8_ electrode exhibited high capacities of 90, 86, 82, 67, and 61 mA h g^–1^ at current densities of 5, 10, 15, 30, and 60 mA g^–1^, which were much higher than those of the bare Mo_6_S_8_ electrode that delivered only 75, 62, 60, 48, and 33 mA h g^–1^ at the same currents of 5, 10, 15, 30, and 60 mA g^–1^, respectively. This is due to the Na_3_PS_4_ coating that improved the reaction kinetics and also minimized the interfacial resistance from 1125 to 654 Ω. Furthermore, Na_3_PS_4_ plays an intermediate role in the chemical compatibility with the sulfide-based cathode, and no further chemical reactions occurred in the Na_3_PS_4_–Mo_6_S_8_ interface.

In another work, Wen et al. developed a coating layer on the surface of the β′′-Al_2_O_3_ solid electrolyte comprising microsized and cotton-cloth-derived disordered carbon tubes (DCT).^108^ The coating over the solid electrolyte effectively reduced interfacial resistance. An improved sodium wetting of the DCT-modified solid electrolyte enables uniform and fast Na ion transport across the interface. The coating significantly reduced the interfacial resistance from 750 to 150 Ω cm^–2^ as compared to that of the symmetrical cell. Even after 400 cycles, the interfacial resistance rose to ∼200 Ω, whereas the cell without a DCT coating exhibited a high interfacial resistance of ∼2000 Ω.

### Employment of a Wetting Agent

Ionic liquid and organic liquid electrolytes have also been used as wetting agents in addition to solid composite electrodes to improve the intimate contact between the cathode and solid electrolyte. The ionic liquid is preferred because of its nonflammability, nonvolatility, high thermal stability, and electrochemical stability since the liquid electrolyte is unstable at high temperatures. Gu et al. proposed a strategy of adding a small amount of liquid electrolyte (5 μL of 0.8 M NaPF_6_ salt in ethylene carbonate-dimethyl carbonate (EC-DMC)) and a nonvolatile and nonflammable ionic liquid (i.e., *N*-methyl-*N*-propylpiperidinium-bis(fluorosulfonyl) imide (PP_13_FSI, ∼5 μL cm^–2^)) as a wetting agent and achieved a significantly reduced interfacial resistance as shown in Figure 10(h and i).^109^ For the AS^3^B configuration, Na_3_V_2_(PO_4_)_3_/liquid electrolyte/Na_3.3_Zr_1.7_La_0.3_Si_2_PO_12_/Na and Na_3_V_2_(PO_4_)_3_/ionic liquid/Na_3.3_Zr_1.7_La_0.3_Si_2_PO_12_/Na batteries with different loadings of cathode materials (1.67 and 3.33 mg cm^–2^) were tested for AS^3^B. The interfacial resistance of Na_3_V_2_(PO_4_)_3_/LE/Na_3.3_Zr_1.7_La_0.3_Si_2_PO_12_/Na was much lower than that of Na_3_V_2_(PO_4_)_3_/Na_3.3_Zr_1.7_La_0.3_Si_2_PO_12_/Na. However, Na_3_V_2_(PO_4_)_3_/ionic liquid/Na_3.3_Zr_1.7_La_0.3_Si_2_PO_12_/Na exhibited interfacial resistance comparable to that of conventional liquid electrolytes.^30,112^

The inclusion of ionic liquid in the cathode materials will facilitate faster Na^+^ transport via the space–charge layer and the electric field induced by the ionic liquid layer. The as-fabricated AS^3^B delivered initial charge and discharge capacities of 116 and 113 mA h g^–1^, respectively, at a current of 0.2 C. The specific discharge capacities were 113, 112, 109, 106, 103, 97, 91, and 86 mA h g^–1^ at cycling rates of 0.2, 0.5, 1, 2, 4, 6, 8, and 10 C, respectively, as shown in Figure 10(j and k). The excellent electrochemical performance demonstrated by Na_3_V_2_(PO_4_)_3_/ionic liquid/Na_3.3_Zr_1.7_La_0.3_Si_2_PO_12_/Na-based AS^3^B was attributed to the high σ of the composite solid electrolyte and the addition of a small amount of ionic liquid at the electrolyte–electrode interface. In another reported work, Jung and Hong et al. obtained AS^3^B by employing an Na_3_SbS_4_-coated NaCrO_2_ cathode with a coating thickness of 200 nm. The as-fabricated Na_3_SbS_4_-coated NCO/Na–Sn cell successfully delivered a discharge capacity of 108 mA h g^–1^, like that of the liquid-electrolyte cell.^110^

### Formation of an Interlayer between Electrode–Electrolyte Interfaces

It has been demonstrated that creating an interlayer or interphase for the ions to be transported between solid electrolytes and cathodes can lower the interfacial resistance. Similar to the liquid electrolyte–Na metal system, SSE also forms three types of interlayers. Type 1 is the thermodynamically stable interface, which does not undergo electrochemical or chemical reactions. Type 2 is the nonpassivizing layer with mixed electronic and ionic conducting pathways (MCI), and type 3 is a stable solid electrolyte interface (SEI), which provides a favorable interface with a high σ and negligible electronic conductivity. Types 1 and 3 are highly recommended for the stable performance of the solid state sodium metal battery. Type 2 results in detrimental dendritic growth, which causes improper contact between the SSE and metal anode. Furthermore, it can be solved by melting sodium metal onto the surface of inorganic SSE. Doping and substitution in the inorganic SSE help to improve the cycling efficiency and also promote the affinity toward sodium metal. Another viable technique is to mitigate the interlayer compatibility by constructing electronically insulating artificial SEI on the surface of the Na-metal anode.^113^

**Figure 11:**
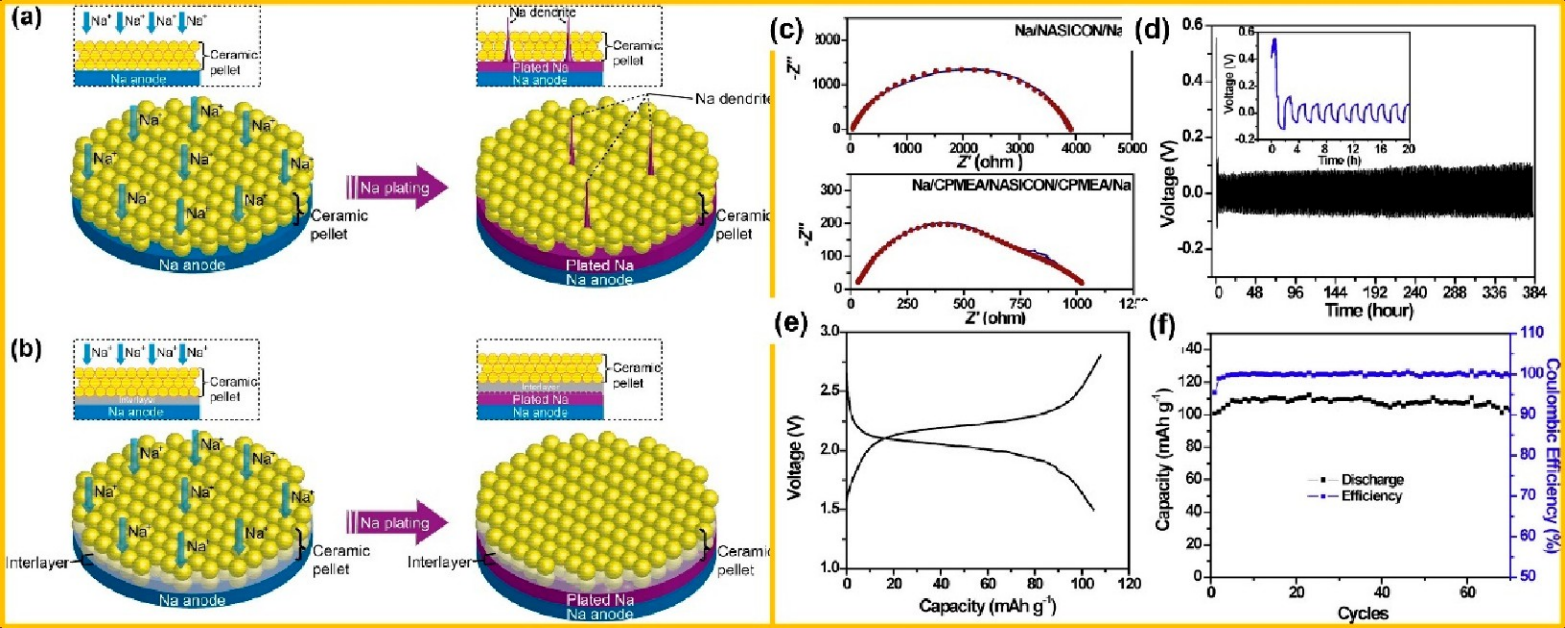
Schematic diagram showing the contact model of IE and the Na metal-based anode: (a) a poor wetting ability ceramic pellet and (b) a good wetting ability artificial interlayer during the plating of sodium. (c and d) Nyquist plots of the device with configuration Na/NASICON/Na and Na/H-NASICON/Na symmetric cell, respectively, at 65 °C. (e) Capacity versus voltage curve of a Na/H-NASICON/gold foil at a scanning rate of 0.5 mV s^–1^ and (f) cycling stability test of the Na/H-NASICON/Na symmetric cells at 65 °C. Figure 11 is reproduced from ref (102). Copyright 2017 American Chemical Society.

Interfacial issues in AS^3^B during charge–discharge cycles may be extensively overcome by introducing a stable interstitial interlayer that is wetted by the anode and should be a good Na-ion conductor. In this direction, Goodenough et al. proposed an AS^3^B with a dendrite-free anode with a reduced anode/ceramic interfacial resistance by the introduction of an in situ-formed thin Na^+^ conductive interfacial layer and an interstitial dry polymer film at the interface. The formation of an interlayer results in the suppression of dendrite growth because the grain boundaries associated with ceramic pellets are not in direct contact with the sodium anode, as depicted in Figure 11(a and b).^114^ The Na/Na symmetrical cell fabricated with a heated Na_3_Zr_2_Si_2_PO_12_ (H-Na_3_Zr_2_Si_2_PO_12_) IE (i.e., Na/H-Na_3_Zr_2_Si_2_PO_12_/Na) exhibits an interfacial resistance from 4000 to 400 Ω cm^–2^ as compared to Na/Na_3_Zr_2_Si_2_PO_12_/Na. Further effective dendrite suppression was assessed by using time-dependent voltage profiles under a current density of 0.15 mA cm^–2^ and then increasing to 0.25 mA cm^–2^. In this context, Na/H-Na_3_Zr_2_Si_2_PO_12_/Na demonstrated stable sodium plating–stripping cycles for up to 550 h, but Na/Na_3_Zr_2_Si_2_PO_12_/Na displayed a short circuit within 1 h when subjected to a current density of 0.15 mA cm^–2^ due to the rapid formation of dendrites, causing a penetration of the grain boundaries as depicted in Figure 11(c–f). This is attributed to the excellent wetting of the interlayer that delivers a more uniform sodium flux across the cathode–electrode interface. On the other hand, an interlayer of cross-linked poly(ethylene glycol) methyl ether acrylate (CPMEA) was introduced on both sides of the NASICON electrolyte in the symmetrical cell with a configuration of Na/CPMEA/H-Na_3_Zr_2_Si_2_PO_12_/CPMEA/Na.^102^ The as-prepared symmetric cell exhibits a stable voltage profile for up to 380 h when subjected to a current density of 0.20 mA cm^–2^, revealing the growth suppression of dendrites.

## Anode–Electrolyte Interface

Owing to the low melting point and mechanical modulus, a sodium metal anode is highly recommended to develop wettable surfaces. Despite such advantages, further progress of AS^3^B is plagued by the issue of high interfacial charge transfer resistance and poor interfacial stability.^115,116^ Moreover, the high chemical reactivity of sodium metal across the anode–electrolyte interface raises a serious concern for developing AS^3^B. In particular, in AS^3^B, an ideal surface is characterized by the interface between the Na anode and solid electrolytes, facilitating rapid ion transport with sacrificial dendrite formation during the plating/stripping processes. Over several charge/discharge cycles, insignificant volume change in the sodium metal anode does not provide any intimate contact across the rigid electrolyte interface, causing a decay in the cyclic stability performance of the AS^3^B. Thus, strategies focusing on the interfacial modifications (structure stabilization, electrolyte optimization, improving contact by mechanical methods, and interface engineering via chemical methods) across the anode–electrolyte interface are highly urged to overcome the existing IE’s to improve the electrochemical and device performance of AS^3^B.^117^ The strategies to improve the anode–electrolyte interfacial contact are as follows:

### Structure Stabilization

Sodium alloy-based anodes are attracting immense attention owing to their high gravimetric (volumetric) specific capacities and adequate Na inserting potential, qualifying them as potential anode candidates for high-energy AS^3^B. The Na alloy as compared to Na metal anodes serves as a better candidate for AS^3^B.^115,117^ The host for the Na metal anode is primarily required to bear the volume change during the plating/stripping processes, as the Na anode is rigid and unable to deform after considerable volume change. A Na alloy, on the other hand, could act as an electron and ion-conductive host because its framework can provide continuous pathways for transporting electrons and Na ions, allowing for Na stripping and plating as well as maintaining seamless interface contact with SSEs, resulting in a morphology bereft of dendrites.

Furthermore, due to their lower reactivity with SSEs compared to that of the Na metal, the alloys can form a stable interface. Despite their superior advantages, alloys are prone to irreversible capacity, huge capacity fading, and poor cycling stability. Recently, large emphasis has been placed on developing alloy-based storage materials. These anodes mainly comprise Na with the elements belonging to groups IVA and VA (i.e., Sn, Sb, Ge, Bi, Si, and P) in the periodic table due to their high theoretical specific capacities. The sodiation parameters of widely reported alloys are given in Table 2.

**Table 2:** Sodiation Parameters of Na-Based Alloy Anodes

Alloy system	Alloy binary phase	Theoretical capacity (mA h g^–1^)	Volume expansion (%)	Electrical conductivity (S cm^–1^)
Ge	Ge → NaGe	369	305	∼1 × 10^–2^
Bi	Bi → Na_3_Bi	385	250	
Sb	Sb → Na_3_Sb	660	390	∼2.5 × 10^4^
Sn	Sn → Na_15_Sn_4_	847	420	∼9 × 10^4^
Si	Si → NaSi	954	114	
P	P → Na_3_P	2596	>300	∼1 × 10^–14^

The Ge-based electrodes are widely preferred in rechargeable batteries owing to their impartial volume swelling. The theoretical specific capacity of the Na–Ge binary phase exhibits a large volumetric capacity of 1974 mA h g^–1^, which is much higher as compared to Ge having a volumetric capacity of 369 mA h g^–1^. The volume swelling of Ge is superior to that of Sn, Sb, and P.^118^ Yu et al. prepared multi-core–shell-structured Bi@N-doped carbon-based electrodes to encounter the structural degradation and instability of SEI at the electrode–electrolyte interface during charge/discharge.^119^ The encapsulated Bi spheres encapsulated by a conductive porous carbon shell contributed to the enhanced electrochemical activity and electrical conductivity of AS^3^B. First, the complete encapsulation of Bi nanospheres in carbon precludes volume changes during the charging/discharging, resulting in SEI formation on the outer surface rather than Bi nanoparticles. On the other hand, the nanosized Bi decreases the diffusion length for both ions and electrons, resulting in a better rate capability and power performance. The as-prepared Bi@N-C composite exhibits an outstanding rate capability of 178 mA h g^–1^ at 100 A g^–1^ with a ultralong cycle life of 235 mA h g^–1^ at 10 A g^–1^ after 2000 charge/discharge cycles for AS^3^B.

Lu et al. reported a Na and Na_15_Sn_4_ composite electrode for AS^3^B. It was concluded based on experimental and theoretical calculations that Na_15_Sn_4_ wetted the surface of the Na_1+*x*_Zr_2_Si_*x*_P_3–*x*_O_12_ solid electrolyte.^120^ The introduction of Na_15_Sn_4_ into the Na matrix enhanced the Na^+^ diffusivity on the anode side that suppresses the pore formation at the anode–electrolyte interface. Consequently, the symmetrical composite anode cell demonstrated excellent performance (i.e., a current density of 2.5 mA cm^–2^ and stable galvanostatic cycling for more than 500 cycles at 0.5 mA cm^–2^).^118^ Yue et al. assembled a AS^3^B with a Na–Sn alloy with acetylene black as the anode, Na_3_PS_4_ as SSE, and SSE-coated Mo_6_S_8_ as the cathode, which also resulted in an improved interfacial contact between the SSE and anode.^93^

Cao et al. established a strong bulk-hybrid Na metal anode employing a melt infusion procedure that combined the molten Na with the surface-modified Na_3.4_Zr_2_Si_2.4_P_0.6_O_12_ (NZSP). A conformal SnO_2_ layer was precoated on the surface of the NZSP particles to enhance the affinity with the molten Na. SnO_2_ can react with Na to create a Na–Sn alloy during heating, considerably improving the Na wettability. Across the composite anode, a rapid and continuous channel for the simultaneous transport of electrons and Na is formed. Because of the compact anode configuration, it delivered stable cycling for 700 h at a current density of 1 mA cm^–2^ with a capacity of 5 mA h cm^–2^.^94^

### Contact Improvement

**Figure 12:**
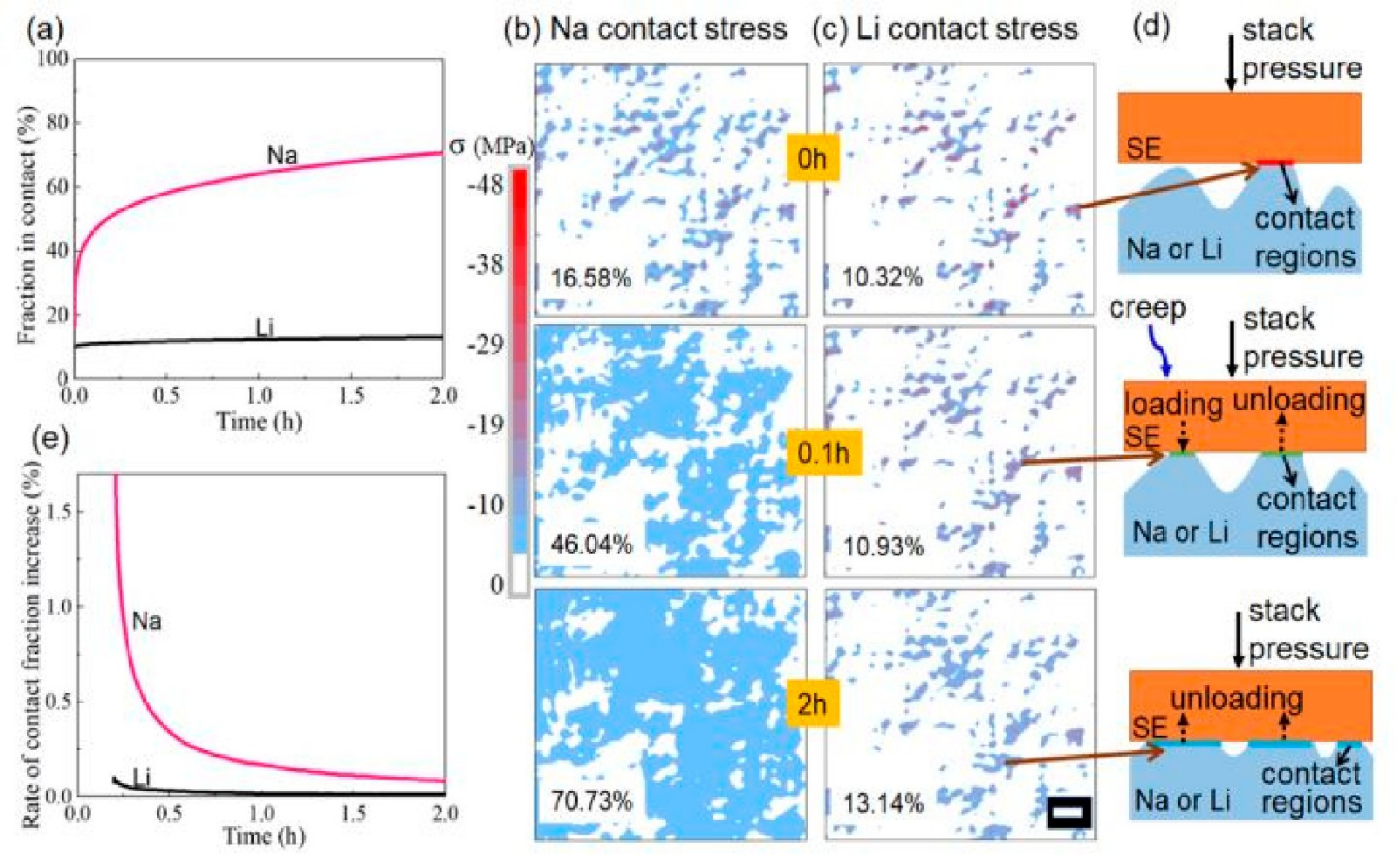
(a) Creep evolution of the sodium–solid electrolyte and lithium–solid electrolyte contact areas with time subjected to a stack pressure of 1.0 MPa. (b) Contact stress maps of the Na surface. (c) Li surface at loading for 0, 0.1, and 2 h. (d) Schematic diagram for the interfacial creep process. (e) Rates of increase in contact fraction with time of the Na-SE and Li-SE interfaces. Figure 12 is reproduced from ref (121). Copyright 2021 American Chemical Society.

Implementing a ceramic electrolyte in AS^3^Bs has the potential to overcome interfacial problems by mechanically restricting the plating of the Na metal from an external pressure, improving uniform and dense deposition and therefore ensuring the stable Na|SSE interfacial contact and high Coulombic efficiency. The mechanical environment is completely different in the context of AS^3^B, where the Na metal is compressed between a rigid metal current collector (Al, Cu, Ni plates, etc.) and SSE. The deformation of Na metal with the use of external pressure plays a crucial role in maintaining low interfacial resistance as the geometry and morphology of a Na metal anode change during charging/discharging (plating/stripping). Recent studies have demonstrated that improving the interface contact and stifling the formation of voids may be accomplished by adding an external pressure (i.e., stack pressure). According to Kasemchainan et al., the constant electrochemical cycling seen in Figure 12(a–e) can be advantageous for Li metal stripping and plating at a stacking pressure of 5–7 MPa. The contact areas of Na and Li interfaces are 70.7 and 13.1% after 2 h of creep. By increasing the loading time, the creeping of the contact regions increases while the noncontact areas also come into contact with the solid electrolyte surface. These new areas start to carry loads and release local stresses to previously loaded areas as shown in Figure 12d. Furthermore, the rate of contact fraction is greater for Na metal than Li metal (Figure 12e).^41^ Zhang et al. proposed a 3D time-dependent model for observing the development of interfaces formed between Na metal and Na-β″-Al_2_O_3_.^99^

The differences due to the contact elastoplasticity are greater than the differences in metal creep effects. A more conformal contact at the high pressure caused by the increased stack pressure may result in less creeping. For example, Uchida et al. have effectively lowered the interfacial resistance of the Na/Na_3_Zr_2_Si_12_PO_12_ (NASICON) assembly to 14 Ω cm^2^ at room temperature using simple mechanical compression. Their work also proved an advantage of the Na/NASICON interface over the Na-β″-Al_2_O_3_ counterpart using the electrochemical impedance technique, which revealed a considerable difference in the activation energies for interfacial charge transfer.^100^

### Electrolyte Optimization

Taking advantage of both inorganic ceramics and organic polymers, the composite electrolytes have been proposed to provide improved σ with high flexibility for reducing the interfacial resistance between solid electrolytes and electrodes.^37,122,123^ A large quantity of research articles have been devoted to composite electrolyte development for an improved interfacial resistance in AS^3^B. For example, Ran et al. developed a low interfacial resistance of around 572 Ω for 40 wt % poly(ethylene oxide) (PEO)-Na_3_Zr_2_Si_2_PO_12_ (NZSP). Therefore, this reduced interfacial resistance delivered good mechanical properties and σ of 4.0 × 10^–5^ S cm^–1^ as well as a good ability to suppress dendrite formation by means of their softer outer layer and the harder middle layer for all-solid-state sodium-ion batteries.^124^

At the same time, Yao et al. showed that polymer PEO with an embedded ceramic conductive β-alumina filler and an interfacial resistance of around 620 Ω possessed excellent interfacial stability, an improved Na-ion transference number, and a flexible electrode–electrolyte interface contact, delivering a high σ of 3.95 × 10^–4^ S cm^–1^ at 60 °C and the widest electrochemical stability window at 5.55 V.^125^ Goodenough et al. made a new revolution by the introduction of a polyethylene glycol diacrylate (PEGDA)/Na_3_Zr_2_Si_2_PO_12_/SCN composite electrolyte membrane with a superior ionic interface of the electrolyte–electrode assembly.^126^ For SSEs, the challenging development issue is improved interfacial resistance. Here, it is about 200 Ω with a good σ of 4.5 × 10^–4^ S cm^–1^. Wu et al. developed a newer polymer–ceramic matrix of PEO with P2-type layered oxide Na_2_Zn_2_TeO_6_ with a nanostructured cathode of Na_2_V_3_(PO_4_)_3_ exhibiting a low interfacial area resistance of 47 Ω cm^2^ and the highest σ of 1 × 10^–3^ S cm^–1^ at 80 °C, which enabled fast sodium-ion transport across the Na/CSE interface.^127^

Recently, Tang et al. were the first to use a polymer–ceramic matrix with a sulfide Na_3_SbS_4_-based inorganic electrolyte, in which they obtained the least interfacial resistance of electrode/electrolyte interface stability and reduced dendrite suppression as well as a σ of 10^–4^ S cm^–1^. Furthermore, sulfide-based polymer composite electrolytes are newer to all-solid-state sodium-ion batteries, and more research on this composite is needed to establish the field for large-scale development. Also, the polymer–ceramic matrix exhibited improved interfacial resistance in all forms of inorganic electrolyte optimization (β-alumina, NASICON, P2-type layered oxide, sulfide-based, etc.) with both high flexibility and improved ionic conductivity in AS^3^B.

### Interface Engineering

To improve the stability of the interface between SSE and Na, different functional interlayers have been investigated. Practically, intimate contact is highly desired in the case of the electrode–electrolyte interface. In addition to the crucial electrolyte threats such as reduced compactness and high electronic conductivity due to grain boundary inorganic electrolytes, dendrite growth is also induced by nonintimate contacts at the interfaces. This can be extensively done by characteristic modification techniques such as atomic layer deposition, the solvent casting method, the wet chemical method, chemical vapor deposition, and applying mechanical pressure in all-solid-state inorganic electrolytes. Furthermore, the overall view to improve the strategies for the electrode–electrolyte interface (cathode–electrolyte/anode–electrolyte) and the techniques and advantages are listed in Table 3.

**Table 3:** Strategies for Improving the Electrode–Electrolyte Interface (Cathode–Electrolyte/Anode–Electrolyte), Techniques and Advantages

Strategy	Techniques	Advantage
Formation of composite cathode	Mixing of active material and electrolyte	High capacity and excellent reversibility, decreased grain boundaries
Employment of wetting agent	Surface coating of ionic liquid	Nonflammability, nonvolatility, high thermal and electrochemical stability
Surface coating	Heating–cooling process	Intimate ionic contact, uniform distributions
Formation of composite electrolyte		Improved ionic conductivity, high flexibility, reduced interfacial resistances
Structure stabilization	Electrochemical plating in the host structure	Capacity controllable
	Thermal molten infusion in the host structure	Commonly used
	Mechanical rolling into the host	Simple and scalable fabrication process
	Nucleation layer on current collector	Uniform deposition
	Na alloy anode	Improved interfacial contact and reduced resistance
Optimization of electrolyte	Composition modification	Composition designable
	Additive	Simple and scalable
	High concentration in organic liquid electrolyte	Stabilized SEI
	Local high-concentration organic liquid electrolyte	Stabilized SEI
Interface engineering	Atomic layer deposition	Uniform deposition
	Solvent casting method	Low-cost and widely used technique
	Wet chemical method	Simple and scalable
	Chemical vapor deposition	Uniform deposition and tunable coating composition
	Applying mechanical pressure	Simple and effective

## Conclusion and Future Outlook

All-solid-state inorganic electrolytes for AS^3^B are a trending aspect in improving battery scenarios, and this class of electrolytes inheriting superior characteristics is capable of outperforming lithium-based battery technology. The inorganic electrolytes of β-alumina (Na-β-Al_2_O_3_) and NASICON (NZPO) exhibit moderate σ and stability at RT. Meanwhile, many dopants were utilized to significantly increase the σ of β-alumina IEs, but such dopants encountered challenges in terms of their sintering temperature, resulting in a lower fractural and densification strength of the electrolyte. Similarly, in NASICON’s solid structure, the substitution of parent atoms produces larger radii and enhances the σ. However, compared to sulfide-IEs, both IEs have low σ values and unstable interfaces in contact with the Na-metal electrode.

Furthermore, the highly conductive inorganic electrolytes employed for AS^3^B are typically chalcogenides, antiperovskites, and borates. Chalcogenides of sulfide-type inorganic solid electrolytes introduce a new glass and glass–ceramic electrolyte. Despite their air stability and handling difficulties, the cubic and tetragonal phases of sulfide-type IEs (Na_3_PS_4_) deliver electrolytes with excellent ionic conductivity, high mechanical strength, excellent electrochemical stability, and better electrode–electrolyte interfaces than other forms of IEs. Likewise, borohydrides (Na_2_B_*n*_H_*n*_) with large cage-like quasi-spherical architectures make several hydrides with alkali ions to form a cost-effective low-temperature method for producing high ionic conductivity. Antiperovskite IEs (e.g., Na_3_OBH_4_) also tend to show high σ and have the advantage of low energy barriers for the characteristic Na-ion transport pathways. P2-type layered sodium-ion conductors (NZTO) exhibit a performance similar to that of NASICON, and the formation of the *P*6_3_22 space phase enables them to exhibit good σ and a better interface between electrode–electrolytes than other oxide-based ISEs.

Overall, chalcogenide-, antiperovskite-, and borohydride-based ISEs are becoming very competitive candidates for large-scale energy storage systems. Much more attention should be paid to the research path of these three IE factors, including salts in electrolytes and their impact upon performance, composition, and structural morphology, dissolution as well as the formation of side products, the effects of temperature and pressure, etc. for the high performance of AS^3^B. Meanwhile, for the large-scale development of AS^3^B, advanced manufacturing techniques such as roll-to-roll processing are expected to be highly studied and adopted due to their ability to efficiently produce dendrite-free interfaces. Furthermore, surface coating techniques such as atomic layer deposition and nanostructured interfaces offer precise control and improved performance at the interface, making them important areas of focus. Additionally, composite electrodes and functional interlayers provide flexibility and enhanced stability, which are also crucial for the long-term reliability of large-scale battery systems. Also, components such as separators, current collectors, binders, etc. have received less attention despite their equal contributions to quality performance. Both electrodes’ structural evolution should be investigated to get a broader idea of specific capacity and capacity retention after cycles. Therefore, developing a Na-based anode along with appropriate cathode materials compatible with electrolytes used in current-generation batteries is crucial to achieving the ultimate position in the growing market.
